# Morphophysiological and Comparative Metabolic Profiling of Purslane Genotypes (*Portulaca oleracea* L.) under Salt Stress

**DOI:** 10.1155/2020/4827045

**Published:** 2020-06-17

**Authors:** Shah Zaman, Muhammad Bilal, Hongmei Du, Shengquan Che

**Affiliations:** ^1^School of Agricultural and Biology, Shanghai Jiao Tong University, Shanghai 200240, China; ^2^School of Life Science and Food Engineering, Huaiyin Institute of Technology, Huaian 223003, China; ^3^School of Design, Department of Landscape Architecture, Shanghai Jiao Tong University, Shanghai 200240, China

## Abstract

Purslane, a fleshy herbaceous plant, plays a pivotal role in various preventive and therapeutic purposes. To date, no report has documented the consequence of salt stress on metabolite accumulation in purslane. Herein, we proposed an insight into the metabolic and physiological traits of purslane under saline stress environments. The gas chromatography-mass spectrometry analysis was used to scrutinize the metabolic profiling of leaves and roots of two purslane genotypes, Tall Green (TG) and Shandong Wild (SD), under the control and saline exposures. Results revealed that the morphological and physiological traits of leaves and roots of both the tested *Portulaca oleracea* cultivars in response to salt stress (100 mM and 200 mM) were dramatically changed. Similarly, significant differences were found in the metabolite profiles among samples under salinity stress treatments as compared with the control. Thorough metabolic pathway analysis, 132 different metabolites in response to 28 days of particular salt stress treatments were recognized and quantified in roots and leaves of purslane, including 35 organic acids, 26 amino acids, 20 sugars, 14 sugar alcohols, 20 amines, 13 lipids and sterols, and 4 other acids. In conclusion, this study can be useful for future molecular experiments as a reference to select gene expression levels for the functional characterization of purslane.

## 1. Introduction

The World Health Organization (WHO) documented purslane (*Portulaca oleracea*) as one of the most important C_4_ medicinal plants, and it was named as “Global Panacea” [1], owing to the presence of immense omega-3 fatty acids and antioxidant vitamins [2]. Purslane can be consumed as a vegetable and play a pivotal role in various preventive and therapeutic purposes particularly in maintaining a healthy immune system and avoiding cardiovascular diseases [3]. Salinity is one of the most vital ecological tasks, which limits plant yield, mostly in the arid and semiarid climates [4]. Salinity in soils and irrigation water is one of the leading abiotic limitations facing agriculture worldwide. An estimated 800 million hectares of agriculture lands are affected globally by salinity [5]. A soil is considered to be saline when the electric conductivity (EC) of the soil solution reaches 4 dS m^−1^ (equivalent to 40 mM NaCl), generating an osmotic pressure of about 0.2 MPa that substantially reduces the crop yield. In addition, salt stress causes necrosis and chlorosis due to the accumulation of Na^+^ that impedes many physiological developments in plants [6].

In plants, most of the salinity adaptation mechanisms involve certain physiological and morphological parameters; the genotypes that cannot grow in high salinity stress are known as glycophytes. On the contrary, halophytes are plants that are able to survive at a high level of NaCl (300-500 mM) due to the development of salt tolerance mechanisms [7]. It is also well recognized that the salt-tolerant genotypes demonstrate increased or unchanged chlorophyll under the salty environments, but chlorophyll contents are decreased in salt-sensitive genotypes [8, 9]. At salt stress conditions, the carotenoid contents are converted from violaxanthin to zeaxanthin by the action of the violaxanthin deepoxidase enzyme [10]. The first most important organ in plants under salt stress conditions is the root system that impairs plant growth in the short term by inducing osmotic stress due to shortened water availability and in the long term by salt-induced ion poisoning due to nutrient disparity in the cytosol [11]. A decrease in the shoot to root ratio or increased root to shoot ratio is a common observation in salt stress regimes, which is connected with water stress rather than salt-induced effects. It is demonstrated that a higher root proportion in a saline environment retains toxic ions in that organ, governing their metabolic and translocation activities into the aerial parts. This response can establish a characteristic plant adaptation mechanism under the saline milieu [12, 13]. In halophytes, salt-resistant plants exhibit the noteworthy capability to complete their life cycle in salt stress conditions. Throughout progression, they might constitute diverse morphological, physiological, and biochemical mechanisms to proliferate the metabolites in environments with high salt concentrations [14].

Several studies have reported on the accumulation of metabolites from plant parts under different salt stress conditions in many crops and found that saline stress exerts differential consequences on the evolution, ion equilibrium, compatible solutes, and metabolism in leaves [15, 16]. Many compatible solutes are nitrogen-derived metabolites, such as amines, amino acids, and betaines; the reason behind this phenomenon is that the availability of nitrogen plays an important role during salinity conditions not only for growth but also for production of these osmoprotectant-related organic solutes [17]. The imbalance between the protein and nitrogen syntheses under salt stress is probably involved in the alterations or increased amino acid level in shoots and roots of plants [17]. In different studies, the salinity treatments exactly increased the levels of proline, sugars, and glycine betaine in wheat [6, 18] like in other Poaceae [19, 20]. It is well documented that most of the studies on wheat under salinity are conducted on leaves; scarce reports are available investigating the effects of salinity on root metabolic profile regarding changes of metabolites associated with cell physiology and root tissues [21, 22].

Reports have shown that studying the effects of salinity in a heterogeneous split root system is more practical than by exposing whole roots to specific levels of NaCl stress [23, 24]. This scenario reproduces the great results during salt stress, which adversely affect the growth, development, and biochemical and physiological mechanisms to acclimatize environmental stress, and various changes occur in the metabolic and physiological reactions in plants during the salinity stress [25]. Previously, we reported that the physiological changes of purslane along with fatty acid contents were increased under 200 mM salinity; however, the effect of the particular salinity stress on metabolite accumulation on purslane remains unknown. Herein, we proposed a new perception of the metabolic and physiological responses of purslane under salinity stress. In the existing research, GC-MS was used to analyze metabolic profiling of leaves and roots of two purslane genotypes, Tall Green (TG) and Shandong Wild (SD), under CK and saline exposure. The physiological and morphological traits were also instantaneously studied. Both types of genotypes were greatly affected under salt stress; mainly, salt stress alters the metabolic mechanism in “SD” roots compared to “TG” under salt stress. This study can be useful for future molecular experiments as a reference to select gene expression levels for the functional characterization of purslane.

## 2. Materials and Methods

### 2.1. Purslane Seeds, Cultivation, and Salt Treatment

To scrutinize the influence of saline treatment on the accumulation of metabolites, two different purslane genotypes were chosen from different geographical locations: “Tall Green” local (“TG”—American origin) and a wild variety “Shandong, China” local (“SD”) (Figure S1). Both genotypes were derived from seeds of a single plant and preserved in laboratory settings through self-fertilization for at least three generations. For the propagation of the seed, 72-cell plastic plug trays (50 cm^3^ per cell) were used. The substrate used was composed of 30% perlite, 40% peat, and 30% vermiculite, and the substrate was supplemented with a sufficient amount of water during the seedling stage. After 14 days, the seedlings with an identical number of leaves and height for “TG” or “SD” were transferred into plastic hydroponic boxes (525 mm × 365 mm × 205 mm) in the greenhouse of School of Agriculture and Biology, Shanghai Jiao Tong University, China, on 23^rd^ March 2018. Plants of both genotypes were treated with three different salt concentrations, i.e., 0 mM, 100 mM, and 200 mM NaCl. For each salt treatment, 12 plants of “TG” and “SD” were set in the same box as one replicate, and experiments were run at least four times. A 15 L quarter strength of Hoagland's solution [26] with an electrical conductivity of 4.0 dS m^−1^ and a pH of 5.8 was put in each plastic box, and a quarter strength of Hoagland's solutions with the same salt concentration was replaced 2 times per week. The plantlets were allowed to grow in a greenhouse with a day and night temperature of 28 ± 2°C and 16 ± 2°C, along with relative moisture and photosynthetically active radiation of 70%-80% and 400 *μ*mol·m^−2^·s^−1^, respectively.

### 2.2. Morphological and Physiological Analysis

At the end of 28 days of salt treatments, the number of leaves, diameter of the stem, main stem length, and root length were recorded with a minimum number of six plantlets. Appropriately, 0.2 g samples of dried roots and leaves were taken, immediately placed in liquid nitrogen, and preserved at -80°C freezer for the physiological and metabolite identification. For Fv/Fm analysis, leaf photochemical efficiency was estimated by measuring chlorophyll fluorescence in the form of the Fv/Fm ratio, with a fluorescence induction monitor (OS 1FL, Opti-Sciences, Hudson, NH). Leaves were covered in a leaf clip to darkness for 30 min before Fv/Fm measurement. For leaf chlorophyll and carotenoid analysis, we cut 0.1 g leaves into pieces, which were placed into small centrifuge tubes with 10 mL dimethyl sulfoxide (DMSO) and saved in the dark environment for 2 to 3 days. After the designated time, the chlorophyll and carotenoid were measured at 663 nm and 645 nm, respectively, by a spectrophotometer (Rochester, NY, USA). Electrolyte leakage was recorded as the percentage of *C*_initial_/*C*_max_ [27].

### 2.3. Metabolite Extraction and Metabolite Profiling Analysis

Leaves and roots of each genotype were harvested separately after the salt treatment and kept at –80°C until further investigation. The polar metabolites were extracted by adopting the protocols as reported earlier with some modifications [27, 28]. Frozen samples were ground into a fine powder with mortars and pestles in liquid nitrogen. Approximately, 25 mg powder of each sample was mixed with 1.4 mL (80% *v*/*v*) aqueous methanol in a 10 mL centrifuge tube. The resultant mixture was centrifuged for 2 h followed by incubation at 70°C in a water bath for 15 min. Afterward, the extracts were centrifuged (at 12000 rpm) for half an hour and the supernatants were decanted into new culture tubes. Following the addition of 0.75 mL of chloroform and 1.4 mL of water, the mixture was vortexed and centrifuged (at 5000 rpm for 5 min), and 300 *μ*L of the polar phase (methanol/water) was dried in a vacuum concentrator. The dried residue was subjected to derivatization in methoxyamine hydrochloride, and *N*-methyl-N-(trimethylsilyl)trifluoroacetamide, and analyzed by GC-MS [29]. The derived extracts were analyzed with a PerkinElmer gas chromatograph coupled with a TurboMass-AutoSystem XL mass spectrometer (Perkin Elmer Inc., Waltham, MA). Adequately, the 1 *μ*L extract was injected into a DB-5MS capillary column (30 m × 0.25 mm × 0.25 *μ*m) with an inlet temperature of 260°C. After a 5 min solvent delay, the initial temperature of the GC oven was maintained at 80°C, which was raised to 280°C with 5°C min^−1^ after 2 min of injection and finally retained at 280°C for 13 min. Helium was employed as the carrier gas with a continuous flow rate of 1 mL min^−1^. The analytical measurements were ensured by using electron impact ionization (70 eV) in the full scan mode (*m*/*z* 30–550).

### 2.4. Statistical Analysis

The Statistical Analysis System (SAS Institute Inc., Cary, NC) recorded comparisons among species and each species' responses to salt stress. Fisher's protected least significant difference (LSD) test was used to assess differences among genotypes and treatment means at the *P* = 0.05 or 0.01 probability level, and the figures displayed were constructed in Microsoft Excel 2016 and SigmaPlot 10.0. For GC-MS analysis, the compounds were identified using TurboMass 4.1.1 software (PerkinElmer Inc.) with online accessible compound libraries (NIST 2011, PerkinElmer Inc., Waltham, USA). SAS version 8.2 was implemented for the statistical analysis of peak areas as reported earlier [30]. The Kyoto Encyclopedia of Genes and Genomes database was used for pathway analysis.

## 3. Results

### 3.1. Morphological and Physiological Traits

**Figure 1:**
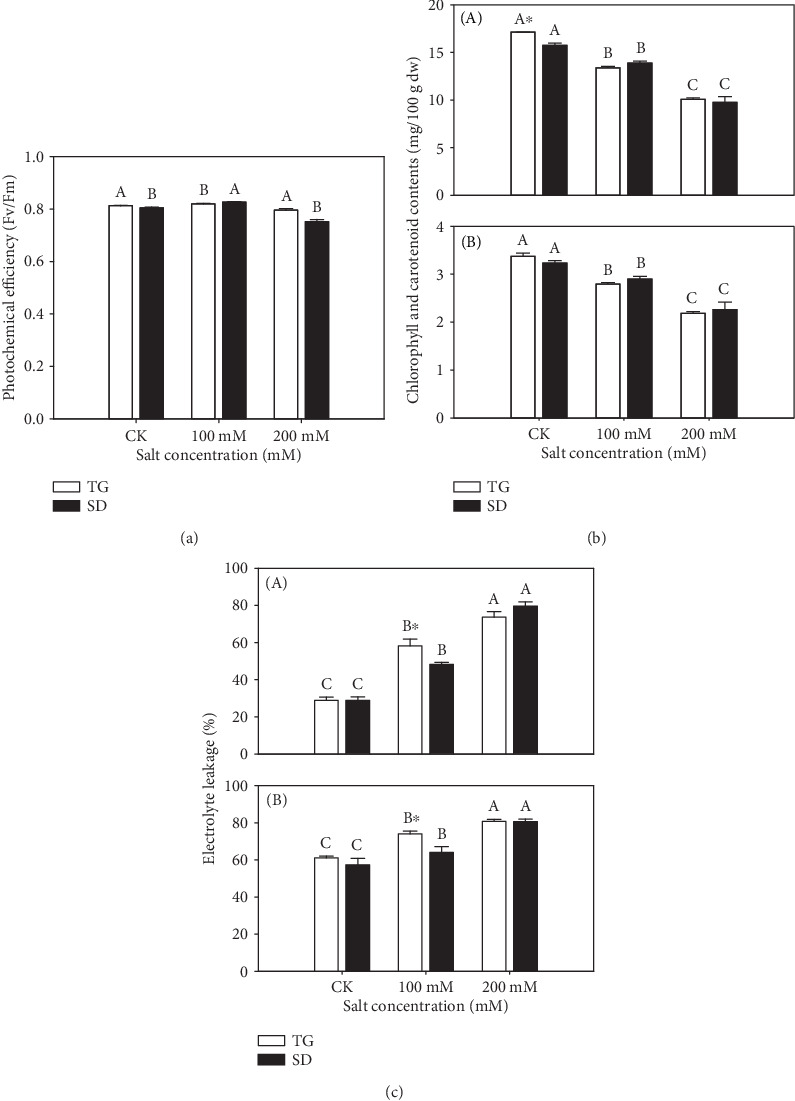
Effects of salt stress on (a) photochemical efficiency (Fv/Fm) in leaves of “TG” and “SD” and (b) chlorophyll and carotenoid content in (A) leaves of “TG” and “SD” (B) roots of “TG” and “SD.” Meanwhile, (c) electrolyte leakage (%) in (A) leaves of “TG” and “SD” and (B) roots of “TG” and “SD” purslane genotypes at 0, 100, and 200 mM salt stress. Vertical bars indicate the SE of each mean (*n* = 4). Columns marked with small letters indicate significant differences between salt treatments for “TG” or “SD” based on the LSD test (*P* = 0.05). Columns marked with a star represent statistical significance for comparison between species at a given NaCl treatment (*P* = 0.05).

After 28 days of the growth period, ANOVA was used to confirm the differences between morphological parameters. There are significant variations observed under particular salt stress in both cultivars.  Table 1 shows that both varieties produced a different number of leaves, length of the stem, diameter of the stem, and length of roots. Salt stress treatment decreased the number of leaves in “TG” at 200 mM and increased in “SD” at the control. The length of the stem was reduced in “SD” at 200 mM and increased in “TG” at the control. The diameter of the stem in “TG” was high at 0 mM and reduced in “SD” at high salinity stress of 200 mM. However, “TG” showed long roots at 100 mM and “SD” showed a short root length at 200 mM compared to the control. Chlorophyll fluorescence measurement is an important index to determine changes in photosynthetic pigments in leaves. The photochemical efficiency (Fv/Fm) values were improved under 0 mM in “TG” and “SD” at 100 mM. Nevertheless, a slight reduction was observed in “SD” at 200 mM (Figure 1(a)). The chlorophyll content was significantly increased under control conditions in “TG” while 200 mM salinity stress decreased the chlorophyll in both cultivars. However, “SD” showed minor changes at 100 mM salt stress compared to “TG.” In addition, the carotenoids were decreased in “TG” and “SD” at 200 mM. Even so, the reduction was observed in “TG” at 200 mM compared to the control. The slight increments of chlorophyll were noticed at 100 mM in “SD.” Moreover, under control condition, both cultivars showed an improvement in carotenoid content (Figure 1(b)). The electrolyte leakage was enhanced with increasing salinity levels in purslane leaves in “SD” at 200 mM and decreased at 100 mM. “TG” showed a significant increment in electrolyte leakage at 100 mM and decreased at 200 mM. Moreover, a substantial decrease was observed at the control in both cultivars. Nevertheless, in roots, the higher increments were observed in both cultivars at 200 mM. However, the salinity stress decreased the electrolyte leakage in “SD” at 100 mM, while an increase was noted in “TG” at 100 mM compared to the control (Figure 1(c)).

### 3.2. Determination of Metabolites from *P*. *oleracea* Leaves and Roots under Different Salinity Stress Conditions

The metabolic variations in roots and leaves of purslane cultivars under the particular condition of salt stress were analyzed by GC-MS to understand the physiological responses and contrast strategies of purslane cultivars to saline stress. Noteworthy differentiations subsist on the metabolite profiles among samples under the salinity stress treatments and control. A total of 132 different metabolites in response to 28 days of salt stress at 0 mM, 100 mM, and 200 mM treatments were identified and quantified in roots and leaves of purslane, mainly including 35 organic acids, 26 amino acids, 20 sugars, 14 sugar alcohols, 20 amines, 13 lipids and sterols, and 4 other acids (Table 2). First, the mean rank of detected metabolites from leaves and roots of both purslane cultivars at given salt stress was computed and the detailed evidence of these metabolites is revealed in Supplementary Tables S1 and S2. In this study, metabolites were filtered based on their relative concentration, and differences in leaves and roots of both cultivars under particular salinity stress conditions were determined. The significant differences based on comparison of statistical values were calculated according to Student's*t*-test (*P* < 0.05, <0.01, and <0.001).

### 3.3. Metabolic Profile in Roots and Leaves of Two *P*. *oleracea* Cultivars in response to Salt Stress

**Figure 2:**
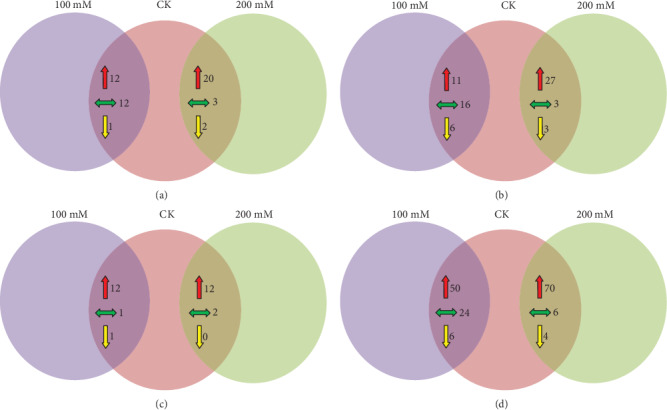
Venn diagrams showing the global comparison of metabolite profile in (a) leaves of “Tall Green,” (b) roots of “Tall Green,” (c) leaves of Shandong Wild, and (d) roots of Shandong Wild purslane after 28 days of salt treatment. A total of 132 compounds were identified by GC-MS, and the numbers in the figure indicate the number of metabolites with a significant up- and downregulation or no fold change. Red and yellow arrows represent the upregulated (>1.5-fold) and downregulated metabolites (<1-fold), respectively, while green arrows represent no fold change.

After the determination of metabolites in response to salt stress, the levels of each metabolite were compared with the control. Based on the result of fold change and significant differences, the levels of metabolite responses were different in roots and leaves in “TG.” In leaves, 12 major metabolites were significantly increased including 2 organic acids, 2 amino acids, 3 sugar alcohols, 3 amines, and 2 lipids and sterols. The other 12 metabolites exhibited no change, and one metabolite named tyramine was decreased at 100 mM. Under salt stress of 200 mM, 20 metabolites increased including 7 organic acids, 6 amino acids, 4 sugar alcohols, and 3 amines. *α*-Linolenic acid and guanosine were meaningfully decreased. In roots, a total of 33 metabolites were selected generally: 11 metabolites increased along with 2 organic acids, 4 amino acid, 2 sugars, 1 sugar alcohol, and 2 lipids and sterols; 16 had no significant change; and 6 were decreased under a salt treatment of 100 mM. Besides, 27 metabolites increased along with 10 organic acids, 7 amino acids, 6 sugar, 2 sugar alcohols, and 2 lipids and sterols at 200 mM salt concentration; three had no difference, and 3 decreased at 200 mM as compared to CK. In “Shandong Wild,” the metabolic responses are dramatically changed in both leaves and roots. In leaves, 12 metabolites including 1 organic acid, 3 amino acids, 1 sugar, 5 sugar alcohols, and 2 amines were increased significantly. In 200 mM salt stress concentration, the 12 metabolites increased including 3 organic acids, 3 amino acids, 1 sugar, and 5 sugar alcohols. Furthermore, 80 metabolites were calculated with a significant fold change in “SD” roots. In 80 filtered metabolites, 50 increased including 20 organic acids, 19 amino acids, 10 sugars, 8 sugar alcohols, 10 amines, and 3 lipids and sterols; 24 had no difference; and 6 decreased significantly at 200 mM. On the other hand, 70 metabolites showed a significant improvement together with 12 organic acids, 14 amino acids, 7 sugars, 7 sugar alcohols, 7 amines, and 3 lipids and sterols. In addition, six metabolites exhibited no changes and 4 were decreased at 100 mM salt concentration compared with the control (Figures 2(a)–2(d)). In TG leaves, L-alanine and L-serine were increased 3.197- and 2.54-fold under 100 and 200 mM salt stress. However, no significant differences were observed in genotype SD under any treatment. While 4-aminobutanoic acid was increased 2.539-fold in genotype TG under 100 and 200 mM and increased 2.095- and 1.899-fold under 0, 100, and 200 mM in genotype SD. On the other hand, L-glutamic acid showed 2.374- and 2.784-fold improvement in TG under 100 and 200 mM, while no changes were observed in SD. L-Glutamine was increased 12.142-fold in TG at 100 and 200 mM salt stress (Supplementary Tables S1 and S2). In roots, L-alanine was increased 2.531-fold at 0 mM and 4.458-fold at 100 and 200 mM. L-Proline showed a 7.859-fold improvement at 200 mM in genotype TG and 22.425-fold at 100 and 200 mM in SD roots, while L-glutamine increased with 117.900-fold in TG roots at 100 mM and 200 mM compared to 0 mM salt stress, respectively. There was no such increment in SD roots.

### 3.4. Total Metabolic Contents in Leaves and Roots of Two *P*. *oleracea* Cultivars under Different Salt Stress Conditions

**Figure 3:**
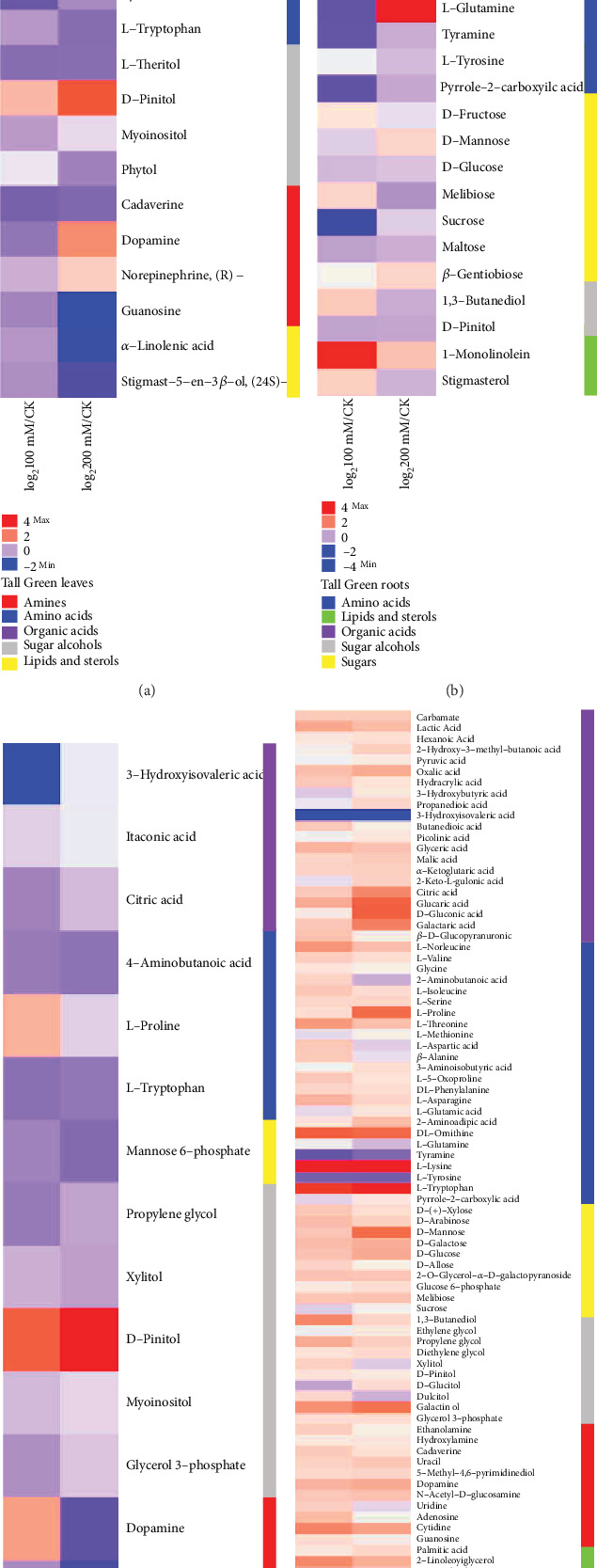
Heat map showing the log_2_ fold change ratios log_2_(treatment/control) for different significant metabolites of (a) leaves of “TG,” (b) roots of “TG,” (c) leaves of “SD,” and (d) roots of “SD” purslane under 0, 100, and 200 mM of salt stress. Fold changes are made in comparison to plants with the control and salt stress conditions, with red representing (max) upregulation and blue (min) representing downregulation.

**Figure 4:**
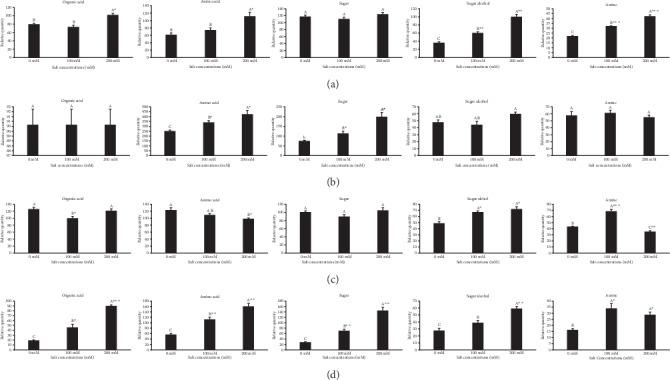
Total relative quantity of organic acids, amino acids, sugars, sugar alcohols, and amines in (a) leaves of “TG,” (b) roots of “TG,” (c) leaves of “SD,” and (d) roots of “SD” purslane under 0, 100, and 200 mM of salt stress. Vertical bars indicate the SE of each mean (*n* = 4). Columns marked with small letters indicate significant differences between salt treatments for “TG” or “SD” based on the LSD test (*P* = 0.05). Columns marked with a star represent statistical significance for comparison between species at a given NaCl treatment (*P* = 0.05).

Major metabolites with fold increase and decrease data were pretreated with formula log_2_(treatment/control), and the R package software was used to construct a heat map, displaying the changes in levels of metabolites between *P*. *oleracea* cultivars in roots and leaves under different salinity stress conditions (Figures 3(a)–3(d)). Further, the total contents of metabolites were prominently changed under different salt concentrations as compared to the control. The total amount of sugar was higher in “TG” under all concentrations, but no meaningful differences were observed. In leaves, the amino acid, organic acid, sugar alcohol, and amine contents were significantly increased at salt stress of 200 mM compared to “CK.” At 100 mM salt stress, high contents of amino acids, sugar alcohols, and amines were accumulated, excluding organic acids and sugars in contrast to the control. In “TG” roots, 200 mM salt stress mainly results in a significant improvement in amino acid and sugar than the control. In addition, no significant changes were observed in organic acid and sugar alcohol (Figures 4(a) and 4(b)).

In leaves of “SD,” the organic acid at 100 mM and the amino acid at 200 mM were decreased significantly relative to the control. No substantial changes were noticed in sugar contents under all tested salt concentrations. However, sugar alcohol was increased significantly at 200 and 100 mM compared with the control. In addition, the total organic acid contents were increased in the roots of “SD” and sugar alcohol was significantly increased at 200 mM. The content of amine enhanced at 100 mM compared to the control. Moreover, the organic acid, amino acid, and sugar contents were also improved significantly at 100 mM and amines at 200 mM. There is no significant change in sugar alcohol compared to the control in the roots of “SD” (Figures 4(c) and 4(d)).

### 3.5. Construction of Metabolic Pathways in between Leaves and Roots of Two *P*. *oleracea* Cultivars under Different Salt Stress Conditions

**Figure 5:**
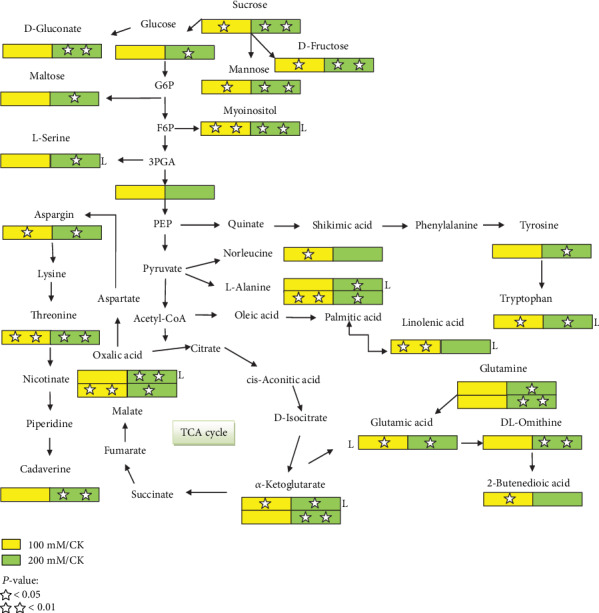
Metabolic pathway showing the log_2_ fold change of identified metabolites in leaves and roots of “TG” purslane. Alphabet “L” represents significant differences of metabolites in leaves, and besides this, all others represent significant differences of metabolites in roots under 0, 100, and 200 mM of salt stress. Square boxes marked with stars represent statistical significance for comparison between species at a given NaCl treatment (*P* = 0.05).

The functions of the identified metabolites in the metabolic pathways were evaluated. Most of the metabolites detected in these pathways are involved in biochemical pathways, such as the TCA cycle, GS/GOGAT cycle, GABA, glycolysis, proline synthesis pathway, shikimic acid, and amino acid metabolic pathway based on search results in the Plant MetaboAnalyst Network and KEGG. One the basis of significant fold increase and decrease, metabolites under different salinity stress conditions were assigned to these metabolic pathways: 14 and 10 in leaves and 33 and 17 in roots of “TG” and, out of 14, 7 in leaves and 80 and 41 in roots of “Shandong Wild.” Some metabolites responded differently to different salt stress conditions in a genotype-dependent manner, such as myoinositol which was increased 1.59- and 2.79-fold at 100 mM and 200 mM salt concentrations compared to the control, respectively, in “TG” leaves. L-Serine, alanine, L-glutamine, cadaverine, *α*-ketoglutaric acid, and malic acid decreased significantly under 100 mM and increased at 200 mM salt stress compared to the control. Tryptophan was increased 1.54-fold at 100 and 1.26-fold at 200 mM. Linolenic acid was increased 1.50-fold at 100 mM and decreased 0.49-fold at 200 mM in comparison to the control. In roots, sugar contents such as sucrose, glucose, fructose, maltose, and mannose were increased 1.72-, 1.54-, 1.97-, 1.23-, and 2.86-fold, respectively, at 200 mM salt concentration compared to the control (Supplementary File S3). No significant fold changes were observed in the sugar level at 100 mM, respectively. In amino acid, L-threonine was increased 1.63- and 2.03-fold under 100 mM moderate and 200 mM high salt concentrations compared to the control, respectively. L-Glutamine increased 6.88-fold at 200 mM, and there is no change observed at 100 mM compared to the control. Inorganic acid D-gluconic, *α*-ketoglutaric, and malic acid were increased 3.85-, 1.07-, and 1.27-fold at 200 mM, and no significant changes were observed at 100 mM. Butanedioic acid was increased 1.08-fold at 100 mM and did not show any change at salt stress of 200 mM relative to the control. 3-Phosphoglycerate was not affected by salt stress as compared to the control (Figure 5).

**Figure 6:**
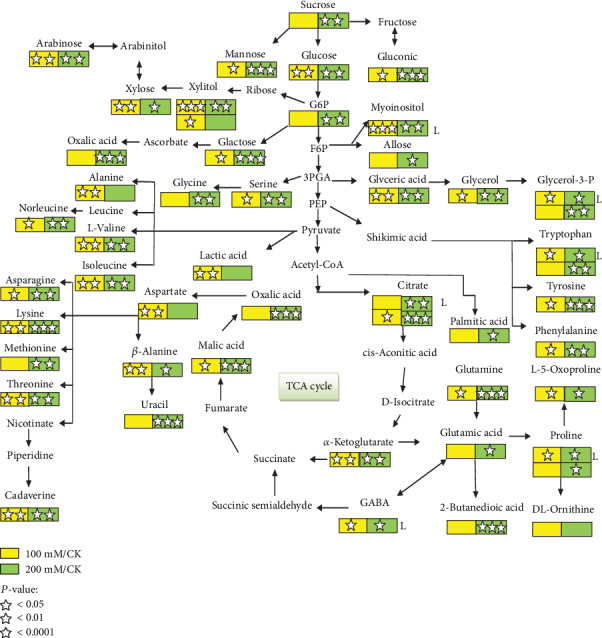
Metabolic pathway showing the log_2_ fold change of identified metabolites in leaves and roots of “SD” purslane. Alphabet “L” represents significant differences of metabolites in leaves, and besides this, all others represent significant differences of metabolites in roots under 0, 100, and 200 mM of salt stress. Square boxes marked with stars represent statistical significance for comparison between species at a given NaCl treatment (*P* = 0.05).

In “Shandong Wild” leaves, the responses of 7 metabolites were significantly increased and decreased under different salt concentrations. Citric acid (1.11), GABA (1.07), proline (3.03), xylitol (1.61), myoinositol (1.67), and glycerol 3-phosphate (1.25) were significantly increased at 100 mM. Additionally, similar metabolites such as myoinositol (1.77), proline (1.72), glycerol 3-phosphate (1.64), and citric acid with (1.57) were increased at 200 mM. Tryptophan, implicated in the shikimic pathway, showed no change under both salinity stress conditions compared to the control. Besides this, in the “Shandong Wild” root, most of the metabolites significantly increased with upregulated fold change at a salt level of 200 mM in contrast to the control. For instance, D-gluconic (4.78), L-proline (4.49), L-lysine (6.21), L-tryptophan (5.89), and D-mannose are more upregulated, and a significant fold change was observed at 200 mM. Moreover, sucrose, citric acid, oxalic acid, butanedioic acid, proline, tryptophan, D-allose, uracil, and palmitic acid were upregulated but were not significantly affected by salt stress compared to the control (Supplementary File S4). On the other hand, a fold increase was observed in the following at 100 mM compared to the control: lactic acid (2.38), glyceric acid (2.07), malic acid (1.15), *α*-ketoglutaric acid (1.26), citric acid (1.54), L-norleucine (2.85), L-valine (1.38), L-isoleucine (1.59), L-serine (1.12), L-threonine (2.73), *β*-alanine (1.50), L-5-oxoproline (1.55), DL-phenylalanine (1.21), L-asparagine (2.05), xylitol (1.34), and cadaverine (1.45). Under 100 mM, sucrose and L-tyrosine resulted in a downregulation in the fold increment and were affected under both salinity stress conditions. Furthermore, DL-ornithine acid showed upregulation but did not demonstrate any significant effect under both salinity concentrations as compared to the control (Figure 6).

## 4. Discussion

In this study, the morphological, physiological, and metabolic changes of the purslane plant were compared under different saline conditions. Results showed that the morphological attributes were affected by 200 mM salt stress compared to 100 mM in “TG.” The number of leaves and roots was decreased at 200 mM than the control, whereas the diameter of the stem and length of roots was reduced at 200 mM in the “SD” wild genotype. During the salinity stress, different abiotic factors such as variations in temperature and nonexistence of O_2_ can reduce the root length and disrupt the natural architecture of the root system because the cell wall of roots under salinity becomes often irregularly thickened and complex. There is a general decrease in plant growth especially in the number of leaves, reduction in root growth through osmosis, and toxic effect in plants subjected to salinity stress [31]. The photochemical efficiency and chlorophyll contents were significantly improved at 0 mM compared to 100 mM salt stress. At a salt level of 200 mM, the chlorophyll content and carotenoid resulted in a decrease in “TG” and “SD.” It is a well-known fact that salt-resistant genotypes showed augmented or unchanged chlorophyll content under the salt stress conditions, whereas the chlorophyll and carotenoid levels declined in salt-sensitive genotypes because of the severity of the salt stress [32, 33]. The leakage of electrolyte was improved with rising salinity levels in purslane leaves in “SD” at 200 mM. Moreover, a substantial reduction was observed at the control in both cultivars. Nevertheless, in roots, the higher increments of electrolyte leakage were observed in both cultivars at 200 mM compared to leaves.

Purslane is among the C_4_ plants with prominent palisade layers on both sides of leaves. C_4_ plants exhibit high water use efficiency and CO_2_ adaptive strategies to make C_4_ photosynthesis, which directly affect chlorophyll pigments during the photosynthesis process in different abiotic stress conditions [34, 35]. Under different salt stress conditions, the cell membrane of the purslane plant plays a key role in sustaining the cell turgor pressure and different physiological attributes. In saline environments, plants elicit diverse biochemical and physiological mechanisms to deal with the resultant stress. These mechanisms comprise alterations in morphology, leaf cell membrane stability, photosynthesis, and biochemical variations [1, 7]. Further, the metabolomics profiles in the roots and leaves of two purslane genotypes were compared under different salt stress conditions. The alteration of plant cells in a salty environment is firmly associated with different metabolic processes [36, 37]. It has been reported that most of the metabolites are involved in different biochemical pathways such as proline synthesis and amino acid pathway metabolism [38]. In these metabolic pathways, carbohydrates, amino acids, and organic acids are key metabolites, which play an important role in plant tolerance and abiotic stress conditions [39]. We found that the contents of organic acid in “TG” leaves significantly increased with increasing salinity and did not show any improvement at 100 mM in contrast to the control (Figure 4(a), leaves). These results indicate that organic acids might engage in regulating intracellular pH by gathering in vacuoles to counteract additional cations [37, 40]. Most of the metabolites involved in organic acids were significant in the roots of “SD” under 200 mM salinity stress (Figure 4(d), roots). The metabolites associated with the TCA cycle may indicate that their metabolic activity is related to the plant's capability to improve its growth under the salt stress environment. An earlier report revealed that many organic acids might function as osmoprotectants and thus possibly improved the barley performance under salt stress [41]. In this experiment, increasing organic acids in roots may act to compensate for charge difference [42]. The salt-induced increase in amino acids in leaves and roots in both genotypes suggests a role for these detected solutes in osmotic adjustment during the physiological and biochemical processes under salt stress mechanisms or might be a common phenomenon of particular genotypes' growth and development during salinity exposure. In addition, the content of amino acids significantly increased in “TG” leaves but significantly decreased in “SD” leaves at 200 mM (Figure 4(a), “TG” leaves; Figure 4(c), “SD” leaves). In roots, the amino acid increment was observed in both of the tested genotypes under 200 mM compared to the control (Figure 4(b), “TG” roots; Figure 4(d), “SD” roots). An increase in tryptophan and phenylalanine content under salinity stress in purslane is linked with shikimic acid and secondary metabolites, which play an essential role in tolerating stress [43]. In amino acid, tryptophan is an inducer of tyrosine and phenylalanine biosynthesis enzymes, which are upregulated in response to abiotic stress [44]. Amino acids such as alanine, valine, threonine, ornithine, glutamine, tyrosine, methionine, and lysine increased significantly under 200 mM salt stress in both genotypes “TG” and “SD,” respectively. Another study confirmed that amino acid metabolism is linked to abiotic stress tolerance [45]. Our results displayed that the sugar contents of purslane seedlings were improved in roots at 200 mM in both genotypes. However, a remarkable decline in sugar content was found in the control and 100 mM (Figure 4(b), “TG” roots; Figure 4(d), “SD” roots). Increasing sugar content in roots of both genotypes under salinity stress acts as an osmolyte to stabilize the integrity of the membrane and maintain cell turgor [46]. High levels of fructose, glucose, sucrose, and maltose have been associated with many plant species under various stress conditions [47, 48]. The increase in proline, threonine, and proline under particular salt stress in “SD” and “TG” roots may be a characteristic phenomenon of genotypes related to salt tolerance. Proline plays an imperative role in plants under salinity by defending plant cell membranes and protein degradation by acting as a ROS capture [49]. In addition, glutamic acid is correlated to chlorophyll biosynthesis and glycine as a precursor of glutathione biosynthesis plays an impotent role in antioxidant defense [50, 51]. Sugar alcohols were increased in “TG” leaves and “SD” roots and leaves at high salt stress (200 mM) relative to the control (Figure 4(a), “TG” leaves; Figures 4(c) and 4(d), “SD” roots). The content of GABA was enhanced in the roots of “SD” under salt stress conditions due to membrane stability and osmotic adjustment [37] in plants under different abiotic factors. Limited studies also confirmed the presence of GABA as noteworthy nonprotein amino acid, and the levels of GABA increased under different environmental stress conditions [52, 53]. Li et al. demonstrated that GABA supports the excess of metabolites in GABA shunt, such as sugar and amino acid metabolism [39], to maintain the metabolic homeostasis under long-term salt stress. In the present case, malate and *α*-ketoglutarate are increased in roots compared to leaves along with citrate at 200 mM in both genotypes. Besides this, the oxalic acid increased significantly in “SD” roots under 200 mM salt stress. Oxalic acid plays a vital role during low temperature and salt stress due to elevated antioxidant capacity in mango and pomegranate. The content of amines was not much higher compared to other metabolites. Even though 200 mM salt stress showed an increase in metabolites in leaves of genotype “TG” (Figure 4(a), leaves), the contradictory increments were observed in “SD” leaves at 200 mM (Figure 4(c), leaves). Fatty acids are the important compatible solutes in the purslane plant, which are located downstream of acetyl-CoA in the metabolism pathway. In our results, the palmitic acid was slightly increased in “SD” at 200 mM, while linolenic acid was increased remarkably in “TG” roots at 100 mM with reference to the control. Our results corroborated that augmentation in linolenic acid plays a crucial role in the tolerance of soybean to salinity stress [47].

## 5. Conclusions

In this study, the metabolic profiling of leaves and roots of two purslane genotypes, Tall Green “TG” and Shandong Wild “SD,” was investigated under the saline stress environments by GC-MS. The morphophysiological attributes of leaves and roots of both the tested *P*. *oleracea* cultivars were dramatically altered following salt stress exposure at 100 and 200 mM. Likewise, significant differences subsist on the metabolite profiles among samples under the salinity stress treatments as compared with the control. Metabolic pathway analysis quantified 132 different metabolites in roots and leaves of purslane in response to particular salt stress treatments including 35 organic acids, 26 amino acids, 20 sugars, 14 sugar alcohols, 20 amines, 13 lipids and sterols, and 4 other acids. Most of the metabolites detected are involved in biochemical pathways, such as the TCA cycle, GS/GOGAT cycle, GABA, glycolysis, proline synthesis pathway, shikimic acid, and amino acid metabolic pathway. In conclusion, this study can be useful for future proteomic and molecular research as a reference to select the gene expression level for functional characterization of purslane, which in turn can be advantageous for sustainable agriculture to meet ever-increasing demands for fresh vegetables.

## Figures and Tables

**Table 1:** Morphological comparison of 2 purslane genotypes “Tall Green” and “Shandong Wild” at 28 d of 0 mM, 100 mM, and 200 mM NaCl stress.

Indexes	“TG”			“SD”		
	0 mM	100 mM	200 mM	0 mM	100 mM	200 mM
Number of leaves	87.4 ± 6.3 a^∗^	79.4 ± 4.0 a	56.8 ± 3.9 b	145.4 ± 6.6 a^∗∗^	143.8 ± 5.6 a	118.4 ± 2.2 b
Length of the stem (cm)	43.8 ± 1.0 a	42.4 ± 0.9 a	37.9 ± 0.8 b	23.6 ± 1.0 a	24.1 ± 0.2 a	21.6 ± 0.3 b
Diameter of the stem (mm)	8.1 ± 0.3 a	8.2 ± 0.2 a	7.4 ± 0.1 b	4.5 ± 0.1 b	4.8 ± 0.0 a	4.2 ± 0.0 c
Length of roots (cm)	20.1 ± 1.7 b	26.6 ± 1.8 a	18.9 ± 0.7 b	13.9 ± 1.5 a	14.7 ± 1.0 a	10.5 ± 0.1 b

^∗^TG + ^∗∗^SD significantly showed a higher number of leaves at 0 mM salt concentration. Values are means, and bars indicate SDs. Columns with different asterisk (^∗∗^) indicate significant difference at *P* < 0.05 (Duncan test).

**Table 2:** List of metabolites identified by gas chromatography-mass spectrometry in leaves and roots of purslane.

No.	RT (min)	Metabolites	Molecular formula	*m*/*z*
		Organic acids		
1	8.177	Carbamate	CH_2_NO_2_^−^	147
2	8.975	Lactic acid	C_3_H_6_O_3_	117
3	9.400	Glycolic acid	C_2_H_4_O_3_	147
4	9.696	Pyruvic acid	C_3_H_4_O_3_	147
5	11.144	Oxalic acid	C_2_H_2_O_4_	190
6	11.331	Hydracrylic acid	C_3_H_6_O_3_	177
7	11.737	3-Hydroxybutyric acid	C_4_H_8_O_3_	88
8	12.908	Propanedioic acid	C_3_H_4_O_4_	147
9	13.075	3-Hydroxyisovaleric acid	C_5_H_10_O_3_	131
10	13.159	*α*-Ketoisocaproic acid	C_6_H_10_O_3_	200
11	14.067	Benzoic acid	C_7_H_6_O_2_	179
12	15.927	Butanedioic acid	C_4_H_6_O_4_	147
13	16.049	Picolinic acid	C_6_H_5_NO_2_	180
14	16.345	Glyceric acid	C_3_H_6_O_4_	189
15	16.654	Itaconic acid	C_5_H_6_O_4_	147
16	16.912	2-Butenedioic acid	C_4_H_4_O_4_	245
17	18.334	Pentanedioic acid	C_5_H_8_O_4_	147
18	18.502	2,4-Dihydroxybutanoic acid	C_4_H_8_O_4_	219
19	19.473	Dihydroxymalonic acid	C_3_H_4_O_6_	73
20	19.943	D-(-)-Citramalic acid	C_5_H_8_O_5_	247
21	20.490	Malic acid	C_4_H_6_O_5_	233
22	22.164	L-Threonic acid	C_4_H_8_O_5_	220
23	22.499	*α*-Ketoglutaric acid	C_5_H_6_O_5_	117
24	23.052	L-(+)-Tartaric acid	C_4_H_6_O_6_	117
25	25.685	2-Aminoadipic acid	C_6_H_11_NO_4_	128
26	26.470	2-Keto-L-gulonic acid	C_6_H_10_O_7_	103
27	27.622	3-Phosphoglycerate	C_3_H_4_O_7_P^−3^	299
28	27.918	Citric acid	C_6_H_8_O_7_	147
29	28.691	Quininic acid	C_11_H_9_NO_3_	255
30	30.467	Glucaric acid	C_6_H_10_O_8_	244
31	31.285	Pantothenic acid	C_9_H_17_NO_5_	201
32	31.381	D-Gluconic acid	C_6_H_12_O_7_	217
33	31.658	Galactaric acid	C_6_H_10_O_8_	73
34	38.159	*β*-D-Glucopyranuronic acid	C_19_H_26_O_8_	217
35	46.463	*cis*-Coutaric acid	C_13_H_12_O_8_	219
		Amino acids		
36	6.896	L-Norleucine	C_6_H_13_NO_2_	56
37	9.735	L-Valine	C_5_H_11_NO_2_	72
38	10.121	L-Alanine	C_3_H_7_NO_2_	116
39	11.621	L-Leucine	C_6_H_13_NO_2_	86
40	12.026	2-Aminobutanoic acid	C_4_H_9_NO_2_	130
41	12.174	L-Isoleucine	C_6_H_13_NO_2_	86
42	14.350	L-Serine	C_8_H_15_NO_5_	132
43	15.348	L-Threonine	C_4_H_9_NO_3_	130
44	15.656	Glycine	C_2_H_5_NO_2_	174
45	16.809	Pyrrole-2-carboxylic acid	C_11_H_16_N_2_O_4_	166
46	18.386	L-Methionine	C_5_H_11_NO_2_S	104
47	18.862	*β*-Alanine	C_3_H_7_NO_2_	174
48	19.596	3-Aminoisobutyric acid	C_4_H_10_ClNO_2_	174
49	21.128	L-5-Oxoproline	C_5_H_7_NO_3_	156
50	21.205	L-Aspartic acid	C_4_H_7_NO_4_	232
51	21.392	4-Aminobutanoic acid	C_4_H_9_NO_2_	174
52	22.582	L-Proline	C_5_H_9_NO_2_	142
53	23.567	L-Glutamic acid	C_5_H_9_NO_4_	246
54	23.599	L-Phenylalanine	C_9_H_11_NO_2_	192
55	26.419	DL-Ornithine	C_5_H_12_N_2_O_2_	186
56	24.629	Asparagine	C_4_H_8_N_2_O_3_	231
57	26.914	L-Glutamine	C_5_H_10_N_2_O_3_	156
58	29.746	Tyramine	C_8_H_11_NO	174
59	29.978	L-Lysine	C_6_H_14_N_2_O_2_	174
60	30.274	L-Tyrosine	C_9_H_11_NO_3_	218
61	35.301	L-Tryptophan	C_11_H_12_N_2_O_2_	203
		Sugars		
62	24.365	D-(+)-Xylose	C_5_H_10_O_5_	103
63	24.855	D-Arabinose	C_5_H_10_O_5_	103
64	25.344	Levoglucosan	C_6_H_10_O_5_	204
65	25.530	D-(-)-Rhamnose	C_16_H_25_N_5_O_15_P_2_	117
66	28.987	D-Fructose	C_6_H_12_O_6_	217
67	36.981	Fructose 6-phosphate	C_6_H_13_O_9_P	299
68	29.270	D-Mannose	C_6_H_12_O_6_	160
69	37.464	Mannose 6-phosphate	C_6_H_13_O_9_P	217
70	29.341	D-Galactose	C_6_H_12_O_6_	205
71	29.495	D-Glucose	C_6_H_12_O_6_	160
72	33.512	D-Allose	C_6_H_12_O_6_	205
73	36.891	2-O-Glycerol-*α*-D-galactopyranoside	C_27_H_66_O_8_Si_6_	204
74	38.327	Glucose 6-phosphate	C_6_H_13_O_9_P	204
75	40.953	D-Lactose	C_12_H_22_O_11_	204
76	42.594	*β*-Gentiobiose	C_12_H_22_O_11_	204
77	43.154	D-(+)-Turanose	C_12_H_22_O_11_	217
78	43.643	Maltose	C_12_H_22_O_11_	204
79	44.886	D-Trehalose	C_12_H_22_O_11_	243
80	48.967	Melibiose	C_12_H_22_O_11_	204
81	54.354	Sucrose	C_12_H_22_O_11_	169
		Sugar alcohols		
82	7.115	Ethylene glycol	C_2_H_6_O_2_	147
83	7.450	Propylene glycol	C_3_H_8_O_2_	117
84	9.548	1,3-Butanediol	C_4_H_10_O_2_	117
85	14.015	Diethylene glycol	C_4_H_10_O_3_	117
86	14.878	Glycerol	C_3_H_8_O_3_	205
87	20.915	L-Threitol	C_4_H_10_O_4_	217
88	25.273	Xylitol	C_5_H_12_O_5_	103
89	28.099	D-Pinitol	C_7_H_14_O_6_	247
90	30.113	D-Glucitol	C_6_H_14_O_6_	205
91	33.164	Myoinositol	C_6_H_12_O_6_	217
92	38.700	Inositol monophosphate	C_6_H_13_O_9_P	299
93	34.716	Phytol	C_20_H_40_O	143
94	35.115	Glycerol 3-phosphate	C_3_H_9_O_6_P	299
95	47.068	Galactinol	C_12_H_22_O_11_	204
		Amines		
96	10.423	Hydroxylamine	H_3_NO	146
97	11.550	Cadaverine	C_5_H_14_N_2_	174
98	14.614	Ethanolamine	C_2_H_7_NO	174
99	16.487	Uracil	C_4_H_4_N_2_O_2_	241
100	18.115	5-Methyl-4,6-pyrimidinediol	C_5_H_6_N_2_O_2_	113
101	20.085	Niacinamide	C_6_H_6_N_2_O	179
102	25.659	Ammelide	C_3_H_4_N_4_O_2_	171
103	26.090	Putrescine	C_4_H_12_N_2_	174
104	27.062	Phosphorylethanolamine	C_2_H_8_NO_4_P	299
105	27.500	9H-Purin-6-ol	C_5_H_4_N_4_O	267
106	32.913	Dopamine	C_8_H_11_NO_2_	174
107	32.933	*N*-Acetyl-D-glucosamine	C_8_H_15_NO_6_	173
108	33.364	Uric acid	C_5_H_4_N_4_O_3_	73
109	34.355	Norepinephrine, (R)-	C_8_H_11_NO_3_	174
110	39.176	Uridine	C_9_H_12_N_2_O_6_	217
111	40.779	2′-Deoxyinosine	C_10_H_12_N_4_O_4_	209
112	41.204	Inosine	C_10_H_12_N_4_O_5_	230
113	42.137	Adenosine	C_10_H_13_N_5_O_4_	230
114	42.858	Cytidine	C_9_H_13_N_3_O_5_	223
115	44.145	Guanosine	C_10_H_13_N_5_O_5_	245
		Lipids and sterols		
116	31.986	Palmitelaidic acid	C_16_H_30_O_2_	117
117	32.392	Palmitic acid	C_16_H_32_O_2_	132
118	35.449	*α*-Linolenic acid	C_18_H_30_O_2_	108
119	35.469	Oleic acid, (Z)	C_18_H_34_O_2_	117
120	35.591	11-Octadecenoic acid, (E)	C_18_H_32_O_4_	117
121	35.945	Stearic acid	C_18_H_36_O_2_	132
122	38.739	Oleamide	C_18_H_35_NO	144
123	41.481	1-Monopalmitin	C_19_H_38_O_4_	203
124	43.392	2-Linoleoylglycerol	C_21_H_38_O_4_	103
125	43.952	1-Monooleoylglycerol	C_21_H_40_O_4_	265
126	44.313	Glycerol monostearate	C_21_H_42_O_4_	203
127	50.820	Stigmasterol	C_29_H_48_O	83
128	51.760	Stigmast-5-en-3*β*-ol, (24S)-	C_29_H_50_O	160
		Others		
129	7.096	Boric acid	BH_3_O_3_	221
130	12.123	Phosphoric acid monomethyl ester	CH_5_O_4_P	163
131	14.331	Urea	CH_4_N_2_O	189
132	14.781	Phosphoric acid	H_3_O_4_P	211

Note: relative retention times (RT, min), molecular formula (MF), and mass ratio (*m*/*z*) of 132 detected metabolites in purslane genotypes “TG” and “SD” at 21 d of 0, 100, and 200 mM NaCl stress.

## Data Availability

All the data used to support the findings of this study are included within the article or in the supplementary materials.

## Supplementary Materials

Supplementary Materials Supplementary Figure S1: (A) “Tall Green” local (“TG”—American origin), (B) a wild variety “Shandong, China” local (“SD”). Supplementary Table S1: metabolites detected by GC-MS from “TG” and “SD” leaves of purslane cultivars at 0, 100, and 200 mM salinity stress. Supplementary Table S2: metabolites detected by GC-MS from “TG” and “SD” roots of purslane cultivars at 0, 100, and 200 mm salinity stress. Supplementary Table S3: Shandong Wild leaves and roots for fold change. Supplementary Table S4: Tall Green leaves and roots for fold change.
