# Supplementary material for: Morphophysiological and Comparative Metabolic Profiling of Purslane Genotypes (Portulaca oleracea L.) under Salt Stress
Source: Biomed Res Int. 2020 Jun 17;2020:4827045. doi: 10.1155/2020/4827045 (PMC7321505; doi:10.1155/2020/4827045)
Supplement: Supplementary Materials — Supplementary Figure S1: (A) “Tall Green” local (“TG”—American origin), (B) a wild variety “Shandong, China” local (“SD”). Supplementary Table S1: metabolites detected by GC-MS from “TG” and “SD” leaves of purslane cultivars at 0, 100, and 200 mM salinity stress. Supplementary Table S2: metabolites detected by GC-MS from “TG” and “SD” roots of purslane cultivars at 0, 100, and 200 mm salinity stress. Supplementary Table S3: Shandong Wild leaves and roots for fold change. Supplementary Table S4: Tall Green leaves and roots for fold change. [file 4827045.f1.zip › 4827045.f1/Metabolomic Table S1-leaves.docx]

Table S1. Metabolites detected by GC-MS from ‘TG’ and ‘SD’ leaves of purslane cultivars at 0, 100 mM and 200 mM salinity stress.

| **Metabolites** | **‘TG’** | | | | |  | **‘SD’** | | |  |
| --- | --- | --- | --- | --- | --- | --- | --- | --- | --- | --- |
|  | 0 mM |  | 100 mM |  | 200 mM | 0 mM | 10 0mM | | | 200 mM |
| **Organic Acids** | Average SE | C% | Average SE | C% | Average SE | Average SE | C% | Average SE | C% | Average SE |
| Carbamic acid | 0.787±0.094b | 0.974 | 0.767±0.117 b | 1.489 | 1.172±0.142 a | 3.177±0.350a | 0.668 | 2.120±0.212b | 0.840 | 2.669±0.190 a |
| Lactic Acid | 11.362±1.023 a | 1.038 | 11.799±1.937 a | 1.121 | 12.733±1.296 a | 14.744±1.175a | 0.794 | 11.712±1.136a | 0.663 | 9.781±0.698b |
| Hexanoic acid | ND | ND | ND | ND | ND | 0.039±0.004a | 0.395 | 0.015±0.002b | 0.595 | 0.023±0.003b |
| Glycolic acid | 0.437±0.019 a | 0.589 | 0.257±0.018 b | 0.992 | 0.434±0.066 a | 0.408±0.020a | 0.592 | 0.241±0.009 b | 0.849 | 0.346±0.039a |
| Pyruvic acid | 0.733±0.028 a | 0.809 | 0.593±0.071 b | 1.234 | 0.905±0.080 a | 1.330±0.129a | 0.512 | 0.681±0.052b | 0.437 | 0.581±0.054b |
| Oxalic acid | 21.750±1.766 a | 0.380 | 8.263±0.655 b | 0.453 | 9.853±0.953 b | 23.961±1.108a | 0.474 | 11.361±1.094 b | 0.563 | 13.501±3.072b |
| Hydracrylic acid | 0.047±0.003 a | 0.520 | 0.024±0.001 b | 0.779 | 0.036±0.005 a | 0.066±0.009a | 0.491 | 0.032± 0.004b | 0.814 | 0.053±0.009 a |
| 3-Hydroxybutyric acid | 0.002±0.000 a | 1.246 | 0.059±0.001 a | 1.059 | 0.002±0.000 a | 0.003±0.002a | 0.796 | 0.002± 0.000a | 1.478 | 0.004±0.001a |
| Propanedioic acid | 0.004±0.004 b | 4.977 | 3.382±0.003 a | 6.330 | 0.028±0.005 a | 0.329±0.071a | 0.399 | 0.132±0.019 b | 1.043 | 0.344±0.023a |
| 3-Hydroxyisovaleric acid | 0.011±0.002 b | 2.261 | 1.862±0.012 b | 26.125 | 0.275±0.035 a | 0.009±0.001b | 0.674 | 0.006±0.001b | 3.680 | 0.035±0.005 a |
| Benzoic Acid | 0.291±0.045 a | 0.202 | 0.059±0.008 b | 0.300 | 0.087±0.022 b | 0.606±0.143a | 0.067 | 0.040±0.003b | 0.118 | 0.071±0.010 b |
| Butanedioic acid | 3.755±0.630 a | 0.901 | 3.382±0.442 a | 1.074 | 4.033±0.183 a | 6.997±0.657a | 0.451 | 3.155±0.252b | 0.778 | 5.443±0.796a |
| Glyceric acid | 1.312±0.105 a | 1.419 | 1.862±0.322 a | 1.211 | 1.590±0.090 a | 2.587±0.240a | 0.275 | 0.711± 0.008b | 0.242 | 0.627±0.086b |
| Itaconic acid | 0.005±0.001 b | 1.761 | 0.008±0.001 a | 2.304 | 0.011±0.001 a | 0.045±0.008b | 3.674 | 0.166±0.018 a | 3.829 | 0.173±0.002a |
| 2-Butenedioic acid | 0.176±0.014 a | 0.516 | 0.091±0.015 b | 0.584 | 0.103±0.017 b | 0.341±0.042a | 0.301 | 0.103±0.011 b | 0.277 | 0.094±0.007b |
| 2,4-dihydroxy-Butanoic acid | 0.083±0.009 a | 0.318 | 0.027±0.005 b | 0.221 | 0.018±0.003 b | 0.173±0.019a | 0.090 | 0.016±0.001b | 0.073 | 0.013±0.001 b |
| Dihydroxymalonic acid | 0.180±0.024 a | 0.887 | 0.160±0.031 a | 0.457 | 0.082±0.020 b | 0.428±0.049a | 0.259 | 0.111± 0.035b | 0.428 | 0.183±0.020b |
| D-(-)-Citramalic acid | 3.227±0.596 a | 1.209 | 3.901±0.383 b | 0.107 | 0.344±0.133 b | 4.324±0.498a | 0.194 | 0.838±0.165b | 0.164 | 0.708±0.253b |
| Malic acid | 17.379±0.757 b | 1.061 | 18.442±3.840 a | 2.190 | 38.063±4.071 a | 28.769±2.006a | 0.623 | 17.933±0.597b | 0.643 | 18.506±1.073b |
| L-Threonic acid | 0.374±0.027 a | 0.611 | 0.229±0.032 b | 0.307 | 0.115±0.014 c | 1.034±0.146a | 0.296 | 0.306± 0.043b | 0.142 | 0.147±0.017b |
| α-Ketoglutaric acid | 0.020±0.004 b | 1.969 | 0.039±0.003 b | 7.337 | 0.147±0.022 a | 0.193±0.008a | 0.768 | 0.148± 0.010a | 0.808 | 0.156±0.028a |
| L-(+)-Tartaric acid | 0.446±0.065 a | 0.670 | 0.299±0.053 a | 0.499 | 0.223±0.025 b | 0.637±0.082a | 0.362 | 0.231± 0.004b | 0.300 | 0.191±0.016 b |
| 2-Keto-l-gluconic acid | 0.309±0.052 a | 0.491 | 0.152±0.015 b | 0.381 | 0.118±0.016 b | 0.654±0.082a | 0.381 | 0.249±0.032 b | 0.383 | 0.251±0.038 b |
| 3-phosphoglycerate | 0.222±0.022 a | 0.684 | 0.152±0.020 b | 0.502 | 0.112±0.007 b | 0.289±0.054a | 0.134 | 0.039±0.015 b | 0.204 | 0.059±0.036 b |
| Citric acid | 4.292±0.522 a | 1.491 | 6.397±1.531 a | 0.875 | 3.756±0.423 a | 12.648±0.894b | 2.165 | 27.381±5.235a | 2.967 | 37.531±2.410 a |
| Quininic acid | 0.154±0.017 a | 0.393 | 0.060±0.006 b | 0.266 | 0.041±0.004 b | 0.058±0.009a | 0.413 | 0.024±0.001b | 0.451 | 0.026±0.002b |
| Glucaric acid | 0.049±0.010 b | 1.346 | 0.066±0.011 b | 2.813 | 0.139±0.009 a | 0.111±0.017a | 0.596 | 0.066± 0.007 b | 0.603 | 0.067±0.006b |
| Pantothenic acid | 0.036±0.002 a | 1.015 | 0.037±0.005 a | 1.266 | 0.046±0.002 a | 0.046±0.004a | 1.168 | 0.054±0.003 a | 1.160 | 0.053±0.007a |
| D-Gluconic acid | 2.241±0.272 a | 0.688 | 1.541±0.111 b | 0.715 | 1.602±0.135 b | 1.808±0.177b | 0.706 | 1.276± 0.117b | 1.576 | 2.850±0.413a |
| Galactaric acid | 5.206±0.618 b | 1.204 | 6.268±0.885 b | 2.380 | 12.392±1.134 a | 7.540±0.398b | 0.875 | 6.596± 0.187c | 1.188 | 8.955±0.139a |
| β-D-Glucopyranuronic acid | 0.107±0.017 a | 0.558 | 0.060±0.007 b | 0.530 | 0.057±0.003 b | 1.255±0.149a | 0.548 | 0.688± 0.081b | 0.953 | 1.197±0.148 a |
| cis-Coutaric acid | 4.480±0.609 b | 1.789 | 8.057±1.870 b | 2.990 | 13.397±0.096 a | 11.364±0.756b | 1.178 | 13.391±0.894b | 1.460 | 16.590±1.166a |

| **Metabolites** |  | **‘TG’** | | | | | | |  | **‘SD’** | | |  |
| --- | --- | --- | --- | --- | --- | --- | --- | --- | --- | --- | --- | --- | --- |
|  | 0 mM | |  | 100 mM |  | | 200mM | 0 mM |  | 100 mM 200 mM | | | |
| **Amino acids** | Average SE | | C% | Average SE | C% | Average SE | | Average SE | C% | Average SE | C% | Average SE | |
| L-Norleucine | 0.115±0.022 a | | 1.405 | 0.161±0.029a | 1.563 | 0.179±0.017 a | | 0.152±0.012a | 0.770 | 0.117±0.030b | 1.409 | 0.214±0.019 a | |
| L-Valine | 3.268±0.344a | | 0.918 | 3.000±0.459a | 1.084 | 3.541±0.156 a | | 3.999±0.545a | 0.808 | 3.230±0.222b | 1.358 | 5.431±0.731 a | |
| L-Alanine | 3.641±0.470 a | | 1.175 | 4.280±0.114b | 3.197 | 11.640±3.066b | | 5.412±0.494a | 0.960 | 5.194±0.665a | 1.061 | 5.742±0.593 a | |
| L-Leucine | 0.398±0.051a | | 0.645 | 0.256±0.033 b | 0.244 | 0.097±0.011 c | | 4.021±0.457 a | 0.278 | 1.116±0.234 b | 0.368 | 1.480±0.160 b | |
| L-Isoleucine | 1.565±0.031a | | 0.706 | 1.105±0.217a | 0.986 | 1.543±0.390 a | | 1.932±0.386 a | 0.514 | 0.993±0.229 b | 0.655 | 1.266±0.139a | |
| L-Serine | 2.013±0.062a | | 1.830 | 3.685±0.675 b | 2.524 | 5.082±0.289 c | | 3.739±0.508 a | 0.872 | 3.259±0.318a | 0.838 | 3.134±0.067a | |
| .L-Threonine | 0.589±0.086b | | 1.244 | 0.732±0.120 a | 1.803 | 1.062±0.124 a | | 0.622±0.021b | 1.999 | 1.244±0.211a | 1.762 | 1.096±0.106 a | |
| Glycin | 0.852±0.038 a | | 0.910 | 0.776± 0.066a | 1.188 | 1.012±0.185 a | | 1.588±0.171a | 0.764 | 1.213±0.111a | 0.500 | 0.793±0.045 b | |
| β-Alanine | 0.415±0.086a | | 0.997 | 0.414± 0.124a | 0.781 | 0.324±0.058 a | | 0.287±0.027a | 0.772 | 0.222±0.046a | 0.588 | 0.169±0.012b | |
| L-5-Oxoproline | 26.279±3.79a | | 1.237 | 32.505±3.679 a | 1.486 | 39.048±4.851a | | 28.458±2.822 a | 0.810 | 23.042±1.53 a | 0.866 | 24.632±0.621a | |
| L-Aspartic acid | 13.131±0.09b | | 1.129 | 14.826±0.938b | 1.945 | 25.540±1.539a | | 35.246±2.157a | 0.645 | 22.726±2.257b | 0.391 | 13.796±1.831c | |
| 4-Aminobutanoic acid | 0.393±0.071b | | 1.285 | 0.505± 0.100b | 2.539 | 0.997±0.221 a | | 4.517±0.757b | 2.095 | 9.463±0.863 a | 1.899 | 8.577±0.162a | |
| L-proline | 0.327±0.061b | | 0.699 | 0.229± 0.040b | 1.695 | 0.555±0.053 a | | 0.189±0.031 b | 8.145 | 1.541±0.294a | 3.296 | 0.624±0.125 b | |
| L-Glutamic acid | 2.209±0.163b | | 2.374 | 5.243± 0.645a | 2.784 | 6.149±0.823 a | | 17.404±1.108b | 1.216 | 21.164±0.511a | 0.946 | 16.459±1.372b | |
| L-Phenylalanine | 1.360±0.127a | | 0.915 | 1.245± 0.234a | 0.590 | 0.803±0.053 b | | 0.628±0.052a | 0.949 | 0.596± 0.059a | 1.830 | 1.149±0.309 a | |
| L-Asparagine | 0.320± 0.038a | | 1.373 | 0.439± 0.103a | 1.569 | 0.502±0.093 a | | 0.518±0.017 b | 1.533 | 0.794±0.113a | 1.149 | 0.595± 0.037a | |
| L-Glutamine | 0.362±0.068b | | 1.456 | 0.527± 0.096b | 12.142 | 4.392±0.542 a | | 6.311±0.788a | 0.604 | 3.812±0.730b | 0.536 | 3.383±0.359 b | |
| Tyramine | 1.880±0.252b | | 0.956 | 1.796±0.175 b | 3.231 | 6.074±1.305 a | | 4.256±0.895a | 1.422 | 6.053±0.359a | 1.283 | 5.462±1.216a | |
| L-Lysine | 1.038± 0.139a | | 1.153 | 1.197± 0.184a | 1.249 | 1.297±0.211 a | | 1.471±0.069a | 0.734 | 1.080±0.146a | 0.797 | 1.172±0.148 a | |
| L-Tyrosine | 1.466± 0.161a | | 0.884 | 1.297± 0.151a | 1.104 | 1.619±0.222 a | | 2.452±0.198 a | 0.877 | 2.149±0.140a | 1.065 | 2.612±0.400 a | |
| L-Tryptophan | 0.020± 0.004a | | 2.959 | 0.058± 0.017a | 2.410 | 0.048±0.013 a | | 0.093±0.016 b | 1.957 | 0.182±0.010 b | 5.563 | 0.517±0.101 a | |

| **Metabolites** |  | | **‘TG’** | | | |  | | **‘SD’** | |  |
| --- | --- | --- | --- | --- | --- | --- | --- | --- | --- | --- | --- |
|  | 0 mM |  | | 100 mM |  | 200mM | 0 mM |  | 100 mM | | 200 mM |
| **Sugars** | Average SE | C% | | Average SE | C% | Average SE | Average SE | C% | Average SE | C% | Average SE |
| D-(+)-Xylose | 0.496±0.059a | 1.117 | | 0.555±0.036a | 0.869 | 0.431±0.023a | 0.305± 0.016a | 1.383 | 0.422±0.037a | 1.344 | 0.411± 0.086a |
| D-Arabinose | 0.957± 0.140a | 0.907 | | 0.868±0.071a | 0.761 | 0.728±0.069 a | 0.958± 0.076a | 0.758 | 0.726±0.069b | 0.832 | 0.797± 0.040a |
| Levoglucosan | 0.178±0.017a | 0.892 | | 0.158±0.019a | 0.689 | 0.122±0.010b | 0.133± 0.019a | 0.919 | 0.122± 0.001a | 0.847 | 0.113± 0.010a |
| D-(-)Rhamnose | 1.524±0.211a | 0.864 | | 1.317±0.226a | 0.707 | 1.077±0.073 a | 0.273± 0.030a | 1.317 | 0.359±0.045a | 1.178 | 0.321± 0.058a |
| D-Fructose | 27.227±2.20b | 0.795 | | 21.635±1.42b | 1.518 | 41.325± 1.568a | 25.532±1.921 a | 1.340 | 34.219±3.344a | 1.406 | 35.899± 5.063a |
| Fructose 6-phosphate | 0.086±0.016a | 0.817 | | 0.070±0.013 a | 0.472 | 0.041± 0.009b | 0.301±0.013 a | 0.171 | 0.052± 0.003b | 0.194 | 0.058± 0.022b |
| D-Mannose | 0.165±0.010a | 0.965 | | 0.159± 0.017a | 0.810 | 0.134± 0.008a | 0.339±0.024 a | 0.494 | 0.167± 0.012b | 0.843 | 0.285± 0.026a |
| Mannose 6-phosphate | 0.056±0.006a | 0.677 | | 0.038±0.008 a | 0.943 | 0.052± 0.006a | 0.005± 0.001b | 2.385 | 0.011± 0.001a | 2.000 | 0.009± 0.001a |
| d-Galactose | 1.524±0.128a | 1.113 | | 1.696± 0.134a | 0.990 | 1.509±0.118a | 4.258±0.466a | 0.807 | 3.434± 0.252a | 0.960 | 4.089± 0.675a |
| D-Glucose | 16.313±0.68b | 0.638 | | 10.404±0.80c | 1.209 | 19.723± 1.310a | 13.431±0.501a | 1.205 | 16.189±1.341a | 1.182 | 15.875± 1.836a |
| D-Allose | 8.240± 0.415a | 1.079 | | 8.893±0.986 a | 0.795 | 6.551± 0.483b | 5.036±0.194a | 0.816 | 4.107± 0.626a | 0.623 | 3.137± 0.603b |
| 2-O-Glycerol-α-d-galactopyranoside | 29.478±1.53a | 1.114 | | 32.843±2.86a | 0.818 | 24.114±0.741b | 23.337±0.898a | 0.587 | 13.707±1.613b | 0.937 | 21.875± 0.378a |
| D-Lactose | 1.340± 0.083a | 1.131 | | 1.515±0.186 a | 0.856 | 1.147± 0.118a | 4.420±0.239a | 0.675 | 2.982± 0.405b | 1.184 | 5.235± 0.502a |
| β-Gentiobiose | 6.174±0.839a | 0.920 | | 5.680±1.232 a | 0.673 | 4.154±0.120a | 4.196± 0.221a | 0.643 | 2.699± 0.200b | 1.007 | 4.225± 0.668a |
| D(+)Turanose | 1.088± 0.198a | 0.057 | | 0.062±0.008b | 0.070 | 0.076±0.023 b | 2.546± 0.387a | 0.208 | 0.531± 0.066b | 0.229 | 0.582± 0.073b |
| Maltose | 1.101±0.208a | 0.522 | | 0.575±0.093 b | 0.556 | 0.612± 0.061b | 0.561± 0.098a | 0.768 | 0.431± 0.091a | 0.749 | 0.420± 0.015a |
| D-Trehalose | 0.252±0.055 a | 0.566 | | 0.142± 0.022b | 0.498 | 0.125±0.004b | 0.189± 0.022a | 0.150 | 0.028± 0.005b | 0.289 | 0.055± 0.006b |
| Melibiose | 19.659±1.04a | 1.188 | | 23.362±1.57a | 1.074 | 21.122±1.724 a | 13.524±0.611a | 0.662 | 8.958± 0.510b | 0.820 | 11.086± 1.333a |
| Sucrose | 1.671±0.387a | 0.271 | | 0.453± 0.047b | 0.715 | 1.194± 0.247a | 1.382±0.056 a | 0.203 | 0.280± 0.017b | 0.295 | 0.407± 0.088b |
| **Sugar alchols** |  |  | |  |  |  |  |  |  |  |  |
| Ethylene glycol | 20.464±1.17a | 0.898 | | 18.385±3.01a | 1.258 | 25.737±3.233a | 24.907±1.073a | 0.689 | 17.169±1.770b | 0.737 | 18.348± 3.308a |
| Propylene glycol | 0.117±0.012a | 1.672 | | 0.195±0.046a | 1.827 | 0.213±0.035 a | 0.065± 0.013b | 2.064 | 0.135± 0.013a | 2.518 | 0.164± 0.025a |
| 1,3- Butanediol | 0.034±0.005 a | 1.029 | | 0.035± 0.002a | 1.470 | 0.050±0.012a | 0.047± 0.012a | 0.667 | 0.031± 0.001b | 1.236 | 0.058± 0.006a |
| Diethylene glycol | 0.049±0.003b | 1.142 | | 0.056± 0.007a | 1.530 | 0.075±0.009a | 0.074± 0.012a | 0.725 | 0.054± 0.004a | 0.998 | 0.074± 0.006a |
| Glycerol | 7.830±0.921a | 1.191 | | 9.327±1.022 a | 0.816 | 6.386± 0.476b | 10.143±0.999a | 0.741 | 7.513± 0.360a | 0.756 | 7.667 ±1.596a |
| L-Theritol | 0.391±0.062b | 1.563 | | 0.612± 0.112b | 2.489 | 0.974± 0.127a | 0.486±0.037 b | 1.540 | 0.749± 0.029a | 1.285 | 0.625± 0.113a |
| Xylitol | 0.335± 0.035a | 0.899 | | 0.301±0.008b | 1.310 | 0.438±0.054 a | 0.275±0.031c | 3.044 | 0.838± 0.025a | 2.471 | 0.680± 0.039b |
| D-Pinitol | 0.449±0.072c | 28.053 | | 12.595±1.49b | 65.811 | 29.547±1.518a | 0.582± 0.075b | 17.329 | 10.091±0.981a | 22.639 | 13.182±2.417a |
| D-Glucitol | 0.736±0.086a | 0.565 | | 0.416± 0.078b | 0.824 | 0.607± 0.119a | 1.354±0.042a | 0.380 | 0.515± 0.033b | 0.251 | 0.340± 0.042c |
| Myo-Inositol | 5.036±0.624c | 3.001 | | 15.116±0.67b | 6.895 | 34.728±3.018a | 8.670± 0.968b | 3.189 | 27.646±0.904a | 3.400 | 29.482± 3.302a |
| Inositol monophosphate | 0.153±0.007b | 1.464 | | 0.223± 0.009a | 0.681 | 0.104±0.008c | 0.302± 0.032a | 0.251 | 0.076± 0.015b | 0.347 | 0.105± 0.024b |
| Phytol | 0.349±0.035c | 8.676 | | 3.024± 0.072b | 2.976 | 1.037±0.138 a | 1.845± 0.338a | 0.904 | 1.669± 0.231a | 0.398 | 0.735± 0.044b |
| Glycerol 3-phosphate | 0.299± 0.009a | 1.000 | | 0.299± 0.053a | 1.319 | 0.395± 0.034 a | 0.231± 0.023b | 2.378 | 0.550± 0.078a | 3.118 | 0.722± 0.139a |

| **Metabolites** | **‘TG’** | | | | |  | **‘SD’** | | |  |
| --- | --- | --- | --- | --- | --- | --- | --- | --- | --- | --- |
|  | 0 mM | C% | 100 mM | C% | 200mM | 0 mM | 100 mM | | | 200 mM |
| **Amines** | Average SE |  | Average SE |  | Average SE | Average SE | C% | Average SE | C% | Average SE |
| Hydroxylamine | 0.021±0.002a | 0.688 | 0.015±0.002a | 1.142 | 0.024±0.006 a | 0.025±0.002a | 0.598 | 0.015± 0.002b | 1.015 | 0.025± 0.004a |
| Cadaverine | 2.540±0.524b | 1.261 | 3.202±0.171b | 2.321 | 5.897±0.517a | 0.197±0.010b | 1.482 | 0.292± 0.020a | 0.441 | 0.087± 0.012c |
| Ethanolamine | 10.905±0.268c | 1.276 | 13.912±0.819b | 1.614 | 17.598±0.820a | 17.859±0.610a | 0.710 | 12.684±1.078b | 0.715 | 12.765±0.325b |
| Niacinamide | 0.078 ±0.003b | 1.390 | 0.109± 0.010a | 1.011 | 0.079±0.007 b | 0.253± 0.014a | 0.519 | 0.132± 0.006b | 0.558 | 0.141± 0.028b |
| Putrescine | 0.997± 0.038a | 0.357 | 0.356± 0.044b | 0.274 | 0.273± 0.047b | 1.547±0.196 a | 0.266 | 0.412± 0.077b | 0.232 | 0.359± 0.069b |
| Phosphorylethanolamine | 0.209± 0.019a | 0.830 | 0.174± 0.012a | 0.767 | 0.160 ±0.016a | 0.232±0.026a | 0.320 | 0.074± 0.012b | 0.860 | 0.200± 0.044a |
| Dopamine | 0.191± 0.013b | 1.814 | 0.347± 0.052b | 33.096 | 6.333± 0.872a | 1.793± 0.092b | 9.730 | 17.444± 2.835a | 1.321 | 2.369±0.218b |
| N-Acetyl-D-glucosamine | 0.005±0.001a | 1.393 | 0.008± 0.001a | 1.564 | 0.009±0.001a | 0.042± 0.005b | 0.709 | 0.030± 0.002b | 1.305 | 0.055± 0.003a |
| Norepinephrine, (R) - | 0.512±0.103b | 4.086 | 2.091±0.071b | 13.534 | 6.927±2.136 a | 14.440±0.878b | 2.339 | 33.781± 1.268a | 1.046 | 15.101±1.532b |
| Uridine | 0.238± 0.072a | 1.372 | 0.326 ±0.063a | 0.927 | 0.220 ±0.007a | 1.537± 0.050a | 0.622 | 0.957± 0.092 b | 0.602 | 0.926± 0.231b |
| Adenosine | 2.017± 0.173a | 1.180 | 2.379± 0.276a | 1.173 | 2.366±0.305 a | 4.524± 0.168a | 0.473 | 2.141± 0.300 b | 0.524 | 2.370± 0.227b |
| Guanosine | 4.213± 0.109b | 2.186 | 9.209± 0.550a | 0.569 | 2.399±0.158 c | 0.798± 0.088a | 0.565 | 0.451± 0.101 b | 0.854 | 0.681± 0.044a |
| **Lipidis and sterols** |  |  |  |  |  |  |  |  |  |  |
| Palmitic Acid | 0.641± 0.073b | 1.577 | 1.010±0.025a | 0.595 | 0.381±0.019 c | 0.362±0.080 a | 0.423 | 0.153± 0.020b | 0.360 | 0.130± 0.010b |
| α-Linolenic acid | 0.173± 0.016b | 2.832 | 0.489±0.044a | 0.715 | 0.123±0.012b | 0.043± 0.009a | 0.406 | 0.017± 0.003 b | 0.569 | 0.025± 0.005a |
| Stearic acid | 0.080± 0.010b | 1.715 | 0.137± 0.007 a | 0.613 | 0.049± 0.003c | 0.080± 0.010a | 0.440 | 0.035± 0.008 b | 0.549 | 0.044± 0.003b |
| Oleamide | 0.257± 0.028b | 1.647 | 0.423± 0.045a | 1.176 | 0.302± 0.061a | 0.222± 0.043a | 0.427 | 0.095± 0.029 b | 0.597 | 0.133± 0.035a |
| 1-Monopalmitin | 0.070 ±0.006a | 1.246 | 0.087 ±0.008a | 0.663 | 0.046±0.002 b | 0.067± 0.005a | 0.332 | 0.022± 0.005b | 0.479 | 0.032± 0.007b |
| Glycerol monostearate | 0.050 ±0.005a | 1.067 | 0.054± 0.005a | 0.648 | 0.033± 0.004b | 0.048± 0.005a | 0.444 | 0.021± 0.002b | 0.320 | 0.015± 0.005b |
| Stigmasterol | 0.046±0.009 a | 1.296 | 0.060±0.011a | 1.228 | 0.057±0.022 a | 0.032± 0.004a | 0.159 | 0.005± 0.002b | 0.184 | 0.006± 0.002b |
| Stigmast-5-en-3β-ol, (24S)- | 0.011± 0.002b | 2.594 | 0.028± 0.005a | 1.195 | 0.013± 0.005b | 0.012± 0.002a | 0.246 | 0.003± 0.001b | 0.275 | 0.003± 0.001b |
| **Others** |  |  |  |  |  |  |  |  |  |  |
| Boric acid | 0.143± 0.007a | 1.412 | 0.201± 0.031a | 0.661 | 0.094±0.000 b | 1.431±0.177a | 0.589 | 0.843± 0.047 b | 1.223 | 1.750± 0.252a |
| Phosphoric acid m ester | 0.236 ±0.022c | 1.585 | 0.374± 0.033b | 2.426 | 0.573±0.056a | 0.524± 0.020a | 0.663 | 0.348± 0.039b | 0.578 | 0.303± 0.021b |
| Urea | 0.618± 0.098b | 2.320 | 1.433± 0.196 b | 11.483 | 7.094±0.822 a | 0.613± 0.149c | 19.414 | 11.906±0.732b | 30.356 | 18.616± 1.278a |
| Phosphoric acid | 74.821±7.729 a | 0.910 | 68.095±13.90a | 0.884 | 66.131±10.04a | 2.501± 0.135a | 0.858 | 2.145± 0.314a | 0.616 | 1.541± 0.434a |

Table showed GC-MS analysis of different metabolites from ‘TG’ and ‘SD’ leaves of purslane cultivars at o, 100 mM and 200 mM stress, Metabolites with a twofold or more than two folds highlighted with red square box AV and SE with ± and ND not detected different letters illustrated significance differences between treatments of detected metabolites, All values are ≤ 0.005.
