# Supplementary material for: Morphophysiological and Comparative Metabolic Profiling of Purslane Genotypes (Portulaca oleracea L.) under Salt Stress
Source: Biomed Res Int. 2020 Jun 17;2020:4827045. doi: 10.1155/2020/4827045 (PMC7321505; doi:10.1155/2020/4827045)
Supplement: Supplementary Materials — Supplementary Figure S1: (A) “Tall Green” local (“TG”—American origin), (B) a wild variety “Shandong, China” local (“SD”). Supplementary Table S1: metabolites detected by GC-MS from “TG” and “SD” leaves of purslane cultivars at 0, 100, and 200 mM salinity stress. Supplementary Table S2: metabolites detected by GC-MS from “TG” and “SD” roots of purslane cultivars at 0, 100, and 200 mm salinity stress. Supplementary Table S3: Shandong Wild leaves and roots for fold change. Supplementary Table S4: Tall Green leaves and roots for fold change. [file 4827045.f1.zip › 4827045.f1/Table S3 Shandong Wild leaves and roots for fold change S 4.docx]

|  | Relative Concentrations | | | | | | | Fold Changes | | |
| --- | --- | --- | --- | --- | --- | --- | --- | --- | --- | --- |
| **Leaves** |  |  |  |  |  |  |  |  |  |  |
| **Metabolites** |  |  |  |  |  |  |  |  |  |  |
| **Organic Acids** | CK  (Resp ratio) | SE | %C | 100 mM  (Resp ratio) | SE | %C | 200 mM  (Resp ratio) | SE | Log_2_^(100 mM /CK)^ | Log_2_^(200 mM/CK)^ |
| 3-Hydroxyisovaleric acid | 0.009 | 0.001 | 674 | 0.006 | 0.001 | 3.680 | 0.035 | 0.005 | -0.58* | 1.96* |
| Itaconic acid | 0.045 | 0.008 | 3.674 | 0.166 | 0.018 | 3.829 | 0.173 | 0.002 | 1.88** | 1.94*** |
| Citric acid | 12.648 | 0.894 | 2.165 | 27.381 | 5.235 | 2.967 | 37.531 | 2.410 | 1.11NS | 1.57** |
| **Amino Acids** |  |  |  |  |  |  |  |  |  |  |
| 4-Aminobutanoic acid | 4.517 | 0.757 | 2.095 | 9.463 | 0.863 | 1.899 | 8.577 | 0.162 | 1.07* | 0.93* |
| L-proline | 0.189 | 0.031 | 8.145 | 1.541 | 0.294 | 3.296 | 0.624 | 0.125 | **3.03*** | 1.72* |
| L-Tryptophan | 0.093 | 0.016 | 1.957 | 0.182 | 0.010 | 5.563 | 0.182 | 0.101 | 0.97* | 0.97* |
| **Sugars** |  |  |  |  |  |  |  |  |  |  |
| Mannose 6-phosphate | 0.005 | 0.001 | 2.385 | 0.011 | 0.001 | 2.000 | 0.009 | 0.001 | 1.14* | 0.85* |
| **Sugar Alcohols** |  |  |  |  |  |  |  |  |  |  |
| Propylene glycol | 0.065 | 0.013 | 2.064 | 0.135 | 0.013 | 2.518 | 0.164 | 0.025 | 1.05* | 1.34* |
| Xylitol | 0.275 | 0.031 | 3.044 | 0.838 | 0.025 | 2.471 | 0.680 | 0.039 | 1.61*** | 1.31** |
| D-Pinitol | 0.582 | 0.075 | 17.329 | 10.091 | 0.981 | 22.639 | 13.182 | 2.417 | **4.12**** | **4.50*** |
| Myo-Inositol | 8.670 | 0.968 | 3.189 | 27.646 | 0.904 | 3.400 | 29.482 | 3.302 | 1.67*** | 1.77** |
| Glycerol 3-phosphate | 0.231 | 0.023 | 2.378 | 0.550 | 0.078 | 3.118 | 0.722 | 0.139 | 1.25* | 1.64* |
| **Amines** |  |  |  |  |  |  |  |  |  |  |
| Dopamine | 1.793 | 0.092 | 9.730 | 17.444 | 2.835 | 321 | 2.369 | 0.218 | **3.28*** | 0.401NS |
| Norepinephrine, (R) – NS2 | 14.440 | 0.878 | 2.339 | 33.781 | 1.268 | 1.046 | 15.101 | 1.532 | 1.23** | 0.06 NS |
| **Lipids and Sterols** | - |  |  | - |  |  | - |  | - | - |
| **Roots** |  |  |  |  |  |  |  |  |  |  |
| **Organic Acids** |  |  |  |  |  |  |  |  |  |  |
| Carbamate | 0.594 | 0.052 | 2.884 | 1.714 | 0.102 | 4.344 | 2.582 | 0.211 | 1.53** | **2.12**** |
| Lactic Acid | 0.594 | 0.056 | 5.184 | 3.082 | 0.262 | 5.449 | 3.239 | 0.472 | **2.38**** | **2.45*** |
| Hexanoic acid | 0.017 | 0.002 | 1.631 | 0.028 | 0.005 | 2.693 | 0.047 | 0.006 | 0.72NS | 1.47* |
| 2-hydroxy-3-methyl-Butanoic acid | 0.014 | 0.002 | 1.503 | 0.021 | 0.002 | 3.818 | 0.054 | 0.005 | 0.58NS | 1.95** |
| Pyruvic acid | 0.594 | 0.024 | 1.410 | 0.838 | 0.050 | 2.369 | 1.408 | 0.191 | 0.50* | 1.25* |
| Oxalic acid | 4.011 | 0.162 | 3.458 | 13.871 | 3.340 | 7.223 | 28.975 | 1.732 | 1.79NS | **2.85***** |
| Hydracrylic acid | 0.062 | 0.011 | 2.967 | 0.184 | 0.005 | 2.647 | 0.164 | 0.021 | 1.57** | 1.40* |
| 3-Hydroxybutyric acid | 3.638 | 0.131 | 0.896 | 3.261 | 0.283 | 2.490 | 9.059 | 1.157 | -0.16NS | 1.32* |
| Propanedioic acid | 0.026 | 0.004 | 1.252 | 0.033 | 0.004 | 3.378 | 0.089 | 0.004 | 0.34NS | 1.78** |
| 3-Hydroxyisovaleric | 3.638 | 0.002 | 0.641 | 0.010 | 0.002 | 2.557 | 0.042 | **0.007** | -8.51NS | -6.44* |
| Butanedioic acid | 3.402 | 0.273 | 2.962 | 10.077 | 0.268 | 2.308 | 7.852 | 0.202 | 1.57NS | 1.21*** |
| Picolinic acid | 0.041 | 0.008 | 1.410 | 0.058 | 0.003 | 2.508 | 0.102 | 0.014 | 0.50NS | 1.31* |
| Glyceric acid | 0.179 | 0.016 | 4.205 | 0.753 | 0.070 | 4.879 | 0.874 | **0.087** | **2.07**** | **2.29**** |
| Malic acid | 1.973 | 0.315 | 2.215 | 4.370 | 0.509 | 4.236 | 8.359 | 0.156 | 1.15* | **2.08***** |
| α-Ketoglutaric acid | 0.088 | 0.012 | 2.410 | 0.211 | 0.011 | 3.732 | 0.327 | 0.020 | 1.26** | 1.89** |
| 2-Keto-l-gluconic acid | 0.033 | 0.005 | 1.172 | 0.038 | 0.004 | 3.703 | 0.121 | 0.004 | 0.20NS | 1.87*** |
| Citric acid | 0.965 | 0.124 | 2.903 | 2.800 | 0.554 | 13.347 | 12.874 | 0.820 | 1.54* | **3.74***** |
| Glucaric acid | 0.019 | 0.003 | 4.860 | 0.092 | 0.014 | 26.097 | 0.495 | **0.015** | **2.28*** | **4.70***** |
| D-Gluconic acid | 0.293 | 0.014 | 1.554 | 0.456 | 0.018 | 27.547 | 8.073 | 0.445 | 0.64** | **4.78***** |
| Galactaric acid | 0.120 | 0.015 | 3.023 | 0.363 | 0.023 | 15.915 | 1.911 | 0.105 | 1.60** | **3.99***** |
| β-D-Glucopyranuronic | 0.779 | 0.072 | 3.150 | 2.454 | 0.150 | 2.370 | 1.846 | 0.172 | 1.66** | 1.24* |
| **Amino Acids** |  |  |  |  |  |  |  |  |  |  |
| L-Norleucine | 0.066 | 0.006 | 7.164 | 0.475 | 0.075 | 5.270 | 0.349 | **0.044** | **2.85*** | **2.40**** |
| L-Valine | 4.369 | 0.447 | 2.600 | 11.360 | 0.909 | 2.959 | 12.930 | 0.782 | 1.38** | 1.57** |
| Glycine | 2.703 | 0.201 | 1.701 | 4.599 | 0.787 | 2.270 | 6.136 | 0.294 | 0.77NS | 1.18** |
| 2-Aminobutanoic acid NS2 | 0.075 | 0.006 | 2.381 | 0.179 | 0.006 | 0.929 | 0.070 | 0.004 | 1.25** | -0.10NS |
| L-Isoleucine | 2.023 | 0.200 | 3.017 | 6.104 | 0.477 | 2.980 | 6.029 | 0.277 | 1.59** | 1.58** |
| L-Serine | 2.520 | 0.243 | 2.174 | 5.480 | 0.933 | 3.406 | 8.585 | 0.676 | 1.12* | 1.77** |
| L-Proline | 1.907 | 0.507 | 1.945 | 3.708 | 0.307 | 22.425 | 42.757 | 7.609 | 0.96NS | **4.49*** |
| L-Threonine | 0.239 | 0.017 | 6.648 | 1.588 | 0.104 | 5.105 | 1.220 | **0.154** | **2.73**** | **2.35**** |
| L-Methionine NS1 | 0.226 | 0.021 | 1.092 | 0.247 | 0.035 | 2.306 | 0.522 | 0.040 | 0.13NS | 1.21** |
| L-Aspartic acid | 5.283 | 0.454 | 2.958 | 15.629 | 0.994 | 1.398 | 7.388 | 0.567 | 1.56** | 0.48NS |
| β-Alanine NS2 | 0.395 | 0.046 | 2.831 | 1.119 | 0.101 | 1.734 | 0.686 | 0.077 | 1.50** | 0.80* |
| 3-Aminoisobutyric acid | 0.044 | 0.005 | 1.443 | 0.063 | 0.009 | 2.956 | 0.129 | 0.014 | 0.52NS | 1.55** |
| L-5-Oxoproline | 8.475 | 0.671 | 2.926 | 24.795 | 2.954 | 2.644 | 22.407 | 2.947 | 1.55* | 1.40* |
| DL-Phenylalanine | 0.975 | 0.110 | 2.315 | 2.258 | 0.231 | 2.859 | 2.788 | 0.207 | 1.21* | 1.52** |
| L-Asparagine | 0.098 | 0.016 | 4.130 | 0.405 | 0.092 | 3.381 | 0.332 | **0.026** | **2.05*** | 1.76** |
| L-Glutamic acid NS1 | 5.050 | 0.339 | 1.070 | 5.403 | 0.498 | 2.475 | 12.497 | 1.899 | 0.10NS | 1.31* |
| 2-Aminoadipic acid | 0.022 | 0.003 | 1.758 | 0.038 | 0.007 | 5.300 | 0.114 | 0.007 | 0.79NS | **2.37**** |
| DL-Ornithine | 0.245 | 0.004 | 1.816 | 4.422 | 0.069 | 17.310 | 5.700 | 0.039 | **4.17NS** | **4.54NS** |
| L-Glutamine | 0.329 | 0.167 | 13.428 | 0.444 | 0.929 | 1.454 | 0.356 | **0.144** | 0.43* | 0.11*** |
| Tyramine | 0.245 | 0.008 | 1.237 | 0.039 | 0.006 | 2.855 | 0.090 | 0.008 | -2.65NS | -1.44* |
| L-Lysine | 0.032 | 0.042 | 3.180 | 1.568 | 0.084 | 4.821 | 2.377 | 0.083 | **5.61**** | **6.21***** |
| L-Tyrosine | 0.493 | 0.005 | 2.041 | 0.106 | 0.015 | 2.889 | 0.150 | 0.005 | -2.22* | -1.72*** |
| L-Tryptophan | 0.052 | 0.090 | 1.734 | 1.623 | 0.252 | 3.307 | 3.094 | 0.246 | **4.96NS** | **5.89**** |
| Pyrrole-2-carboxylic acid | 0.020 | 0.002 | 2.463 | 0.020 | 0.003 | 13.448 | 0.049 | 0.049 | 0.00** | 1.29* |
| **Sugars** |  |  |  |  |  |  |  |  |  |  |
| D-(+)-Xylose | 0.185 | 0.022 | 2.958 | 0.549 | 0.021 | 2.981 | 0.553 | 0.012 | 1.57** | 1.58* |
| D-Arabinose | 0.677 | 0.076 | 3.573 | 2.420 | 0.227 | 3.679 | 2.492 | 0.157 | 1.84** | 1.88** |
| D-Mannose | 0.063 | 0.008 | 3.028 | 0.191 | 0.032 | 22.090 | 1.393 | 0.076 | 1.60* | **4.47***** |
| d-Galactose | 0.172 | 0.025 | 3.605 | 0.618 | 0.087 | 6.964 | 1.195 | 0.059 | 1.85* | **2.80***** |
| D-Glucose | 7.449 | 0.678 | 3.198 | 23.821 | 1.526 | 7.428 | 55.332 | 6.824 | 1.68** | **2.89**** |
| D-Allose | 0.102 | 0.026 | 2.348 | 0.241 | 0.076 | 2.235 | 0.229 | 0.021 | 1.24NS | 1.17* |
| 2-O-Glycerol-α-d-galactopyranoside | 0.942 | 0.076 | 3.173 | 2.988 | 0.384 | 4.587 | 4.319 | 0.295 | 1.67* | **2.20**** |
| Glucose 6-phosphate | 0.084 | 0.007 | 1.583 | 0.133 | 0.017 | 2.864 | 0.241 | 0.017 | 0.66NS | 1.52** |
| Melibiose | 1.112 | 0.178 | 3.111 | 3.460 | 0.535 | 4.888 | 5.435 | 0.895 | 1.64* | **2.29*** |
| Sucrose NS1 | 0.112 | 0.009 | 0.940 | 0.105 | 0.021 | 2.195 | 0.246 | 0.012 | -0.09NS | 1.14** |
| **Sugar Alcohols** |  |  |  |  |  |  |  |  |  |  |
| 1,3-Butanediol | 0.018 | 0.003 | 9.012 | 0.167 | 0.023 | 3.283 | 0.061 | **0.011** | **3.21**** | 1.76* |
| Ethylene glycol | 8.149 | 0.579 | 1.368 | 11.147 | 1.044 | 2.466 | 20.097 | 1.176 | 0.45NS | 1.30** |
| Propylene glycol | 0.038 | 0.005 | 4.889 | 0.183 | 0.031 | 3.870 | 0.145 | **0.020** | **2.27*** | 1.93* |
| Diethylene glycol | 0.013 | 0.002 | 1.755 | 0.023 | 0.002 | 3.001 | 0.040 | 0.003 | 0.82* | 1.62** |
| Xylitol | 0.061 | 0.007 | 2.544 | 0.154 | 0.024 | 1.364 | 0.083 | 0.007 | 1.34* | 0.44NS |
| D-Pinitol | 1.494 | 0.330 | 1.749 | 2.613 | 0.272 | 2.393 | 3.575 | 0.352 | 0.81NS | 1.26* |
| D-Glucitol | 0.279 | 0.063 | 0.529 | 0.148 | 0.020 | 2.989 | 0.835 | **0.034** | -0.91NS | 1.58** |
| Dulcitol NS2 | 0.016 | 0.001 | 2.118 | 0.034 | 0.003 | 0.974 | 0.016 | 0.003 | 1.09* | 0.00NS |
| Galactinol | 0.084 | 0.005 | 7.735 | 0.648 | 0.080 | 19.134 | 1.602 | **0.062** | **2.95**** | **4.25***** |
| Glycerol 3-phosphate | 1.194 | 0.103 | 1.937 | 2.313 | 0.260 | 2.989 | 3.569 | 0.065 | 0.95* | 1.58*** |
| **Amines** |  |  |  |  |  |  |  |  |  |  |
| Ethanolamine | 9.217 | 0.869 | 2.547 | 23.470 | 3.214 | 2.227 | 20.528 | 1.698 | 1.35* | 1.16** |
| Hydroxylamine | 0.011 | 0.001 | 1.727 | 0.018 | 0.003 | 2.614 | 0.028 | 0.003 | 0.71NS | 1.35** |
| Cadaverine | 0.365 | 0.035 | 2.741 | 1.000 | 0.034 | 2.768 | 1.010 | 0.035 | 1.45** | 1.47** |
| Uracil | 0.168 | 0.014 | 2.429 | 0.408 | 0.083 | 4.468 | 0.750 | 0.036 | 1.28NS | **2.16***** |
| 5-Methyl-4,6-pyrimidinediol | 0.015 | 0.002 | 2.147 | 0.032 | 0.006 | 3.486 | 0.052 | 0.003 | 1.09NS | 1.79* |
| Dopamine | 0.092 | 0.015 | 4.127 | 0.381 | 0.040 | 7.474 | 0.691 | **0.085** | **2.05**** | **2.91**** |
| N-Acetyl-D-glucosamine | 0.058 | 0.006 | 3.008 | 0.173 | 0.020 | 5.149 | 0.297 | 0.068 | 1.58* | **2.36*** |
| Uridine | 0.365 | 0.004 | 2.607 | 0.950 | 0.006 | 1.535 | 0.559 | 0.029 | 1.38* | 0.61NS |
| Adenosine | 0.602 | 0.032 | 3.726 | 2.243 | 0.131 | 2.196 | 1.321 | 0.089 | 1.90** | 1.13** |
| Cytidine | 0.017 | 0.004 | 9.791 | 0.164 | 0.006 | 8.841 | 0.148 | **0.029** | **3.27***** | **3.12*** |
| Guanosine NS1 | 0.304 | 0.018 | 1.981 | 0.602 | 0.185 | 2.344 | 0.712 | 0.097 | 0.99NS | 1.23* |
| **Lipids and Sterols** |  |  |  |  |  |  |  |  |  |  |
| Palmitic Acid | 0.378 | 0.026 | 1.651 | 0.624 | 0.101 | 3.339 | 1.261 | 0.191 | 0.72NS | 1.74* |
| 2-linoleoylglycerol | 0.069 | 0.008 | 9.024 | 0.619 | 0.045 | 7.145 | 0.490 | **0.062** | **3.17**** | **2.83**** |
| 1-Monolinolein | 0.008 | 0.001 | 5.113 | 0.043 | 0.003 | 4.580 | 0.038 | **0.009** | **2.43**** | **2.25*** |
| Glycerol monostearate | 0.051 | 0.008 | 3.070 | 0.156 | 0.024 | 1.484 | 0.076 | 0.010 | 1.61* | 0.58NS |

Folds changes were using the formula log_2_ ^(treatment/control)^. *indicate significance 95% (p<0.05) - ** indicate significance 99% (p<0.01) - ***indicate high significance 99.9% (p<0.001)

Table 2. Relative content and fold changes of foremost metabolites detected in leaves and roots of purslane after 22 days of treatments, the relative content of each metabolite in each class is a mean of data from four biological replicates detected by GC-MS. Metabolites with bold numbers indicate greater than or equal to two fold increments and the metabolites less than two folds indicate with underlined numbers in existing class under CK, 0 mM and 200 mM salinity stress.
