# Supplementary material for: Morphophysiological and Comparative Metabolic Profiling of Purslane Genotypes (Portulaca oleracea L.) under Salt Stress
Source: Biomed Res Int. 2020 Jun 17;2020:4827045. doi: 10.1155/2020/4827045 (PMC7321505; doi:10.1155/2020/4827045)
Supplement: Supplementary Materials — Supplementary Figure S1: (A) “Tall Green” local (“TG”—American origin), (B) a wild variety “Shandong, China” local (“SD”). Supplementary Table S1: metabolites detected by GC-MS from “TG” and “SD” leaves of purslane cultivars at 0, 100, and 200 mM salinity stress. Supplementary Table S2: metabolites detected by GC-MS from “TG” and “SD” roots of purslane cultivars at 0, 100, and 200 mm salinity stress. Supplementary Table S3: Shandong Wild leaves and roots for fold change. Supplementary Table S4: Tall Green leaves and roots for fold change. [file 4827045.f1.zip › 4827045.f1/Table S4 Tall Green leaves and roots leaves and roots for fold change S 3.docx]

|  | Relative Concentrations | | | | | | | | Fold Changes | |
| --- | --- | --- | --- | --- | --- | --- | --- | --- | --- | --- |
| **Leaves** |  | |  |  | |  |  | |  | |
| **Metabolites** |  | |  |  | |  |  | |  | |
| **Organic Acids** | CK  (Resp ratio) | SE | %C | 100 mM  (Resp ratio) | SE | %C | 200 mM  (Resp ratio) | SE | Log_2_^(100 mM /CK)^ | Log_2_^(200 mM/CK)^ |
| Propanedioic acid | 0.004 | 0.004 | 4.977 | 0.022 | 0.003 | 6.330 | 0.028 | 0.005 | **9.72*** | **2.81*** |
| 3-Hydroxyisovaleric acid | 0.011 | 0.002 | 2.261NS | 0.024 | 0.012 | 26.125 | 0.275 | 0.035 | **7.40NS** | **4.64**** |
| Itaconic acid | 0.005 | 0.001 | 1.761 | 0.008 | 0.001 | 2.304 | 0.011 | 0.001 | 0.68ns | 1.14** |
| Malic acid | 17.379 | 0.757 | 1.061NS | 18.442 | 3.840 | ^2.2^ 2.190 | 38.063 | 4.071 | 0.09NS | 1.13** |
| α-Ketoglutaric acid | 0.020 | 0.004 | 1.969 | 0.039 | 0.003 | 7.337 | 0.147 | 0.022 | 0.96* | **2.88*** |
| Glucaric acid | 0.049 | 0.010 | 1.346NS | 0.066 | 0.011 | 2.813 | 0.139 | 0.009 | 0.43NS | 1.50* |
| Galactaric acid | 5.206 | 0.618 | 1.204NS | 6.268 | 0.885 | 2.380 | 12.392 | 1.134 | 0.27NS | 1.25* |
| cis-Coutaric acid | 4.480 | 0.609 | 1.789 | 8.057 | 1.870 | 2.990 | 13.397 | 0.296 | 0.85NS | 1.58ns |
| **Amino Acids** |  |  |  |  |  |  |  |  |  |  |
| L-Alanine | 3.641 | 0.470 | 1.175NS | 4.280 | 0.114 | 3.197 | 11.640 | 3.066 | 0.23NS | 1.68ns |
| L-Serine | 2.013 | 0.062 | 1.830 | 3.685 | 0.675 | 2.524 | 5.082 | 0.289 | 0.87ns | 1.34* |
| 4-Aminobutanoic acid | 0.393 | 0.071 | 1.285NS | 0.505 | 0.100 | 2.539 | 0.997 | 0.221 | 0.36NS | 1.34* |
| L-Glutamic acid | 2.209 | 0.163 | 2.374 | 5.243 | 0.645 | 2.784 | 6.149 | 0.823 | 1.25* | 1.48* |
| L-Glutamine | 0.362 | 0.068 | 1.456NS | 0.527 | 0.096 | 12.142 | 4.392 | 0.542 | 0.54NS | **3.60*** |
| Tyramine | 1.880 | 0.252 | **0.956NS** | 1.796 | 0.175 | 3.231 | 6.074 | **1.305** | -0.07NS | 1.69** |
| L-Tryptophan | 0.020 | 0.004 | 2.959 | 0.058 | 0.017 | 2.410 | 0.048 | 0.013 | 1.54* | 1.26* |
| **Sugars** | – |  |  | – |  |  | – |  | – | – |
| **Sugar Alcohols** |  |  |  |  |  |  |  |  |  |  |
| L-Theritol | 0.391 | 0.062 | 1.563 | 0.612 | 0.112 | 2.489 | 0.974 | 0.127 | 0.65NS | 1.32** |
| D-Pinitol | 0.449 | 0.072 | 28.053 | 12.595 | 1.491 | 65.811 | 29.547 | 1.518 | **4.81**** | **6.04***** |
| Myo-Inositol | 5.036 | 0.624 | 3.001 | 15.116 | 0.678 | 6.895 | 34.728 | 3.018 | 1.59** | **2.79**** |
| Phytol | 0.349 | 0.035 | 8.676 | 3.024 | 0.072 | 2.976 | 1.037 | 0.138 | **3.12***** | 1.57** |
| **Amines** |  |  |  |  |  |  |  |  |  |  |
| Cadaverine | 2.540 | 0.524 | 1.261NS | 3.202 | 0.171 | 2.321 | 5.897 | 0.517 | 0.33NS | 1.22** |
| Dopamine | 0.191 | 0.013 | 1.814 | 0.347 | 0.052 | 33.096 | 6.333 | 0.872 | 0.86* | **5.05***** |
| Norepinephrine, (R) - | 0.512 | 0.103 | 4.086 | 2.091 | 0.071 | 13.534 | 6.927 | 2.136 | 2.03** | 3.76* |
| Guanosine | 4.213 | 0.109 | 2.186 | 9.209 | 0.550 | 0.569 | 2.399 | 0.158 | 1.13** | -0.81** |
| **Lipids and Sterols** |  |  |  |  |  |  |  |  |  |  |
| α-Linolenic acid | 0.173 | 0.016 | 2.832 | 0.489 | 0.044 | 0.715 | 0.123 | 0.012 | 1.50** | -0.49ns |
| Stigmast-5-en-3β-ol, (24S)- | 0.011 | 0.002 | 2.594 | 0.028 | 0.005 | 1.195nS | 0.013 | 0.005 | 1.35* | 0.24NS |
| **Roots** |  |  |  |  |  |  |  |  |  |  |
| **Organic Acids** |  |  |  |  |  |  |  |  |  |  |
| Pyruvic acid | 1.249 | 0.061 | 1.089NS | 1.360 | 0.256 | 3.086 | 3.855 | 0.354 | 0.12NS | 1.63** |
| 3-Hydroxybutyric acid | 4.118 | 0.595 | 1.344NS | 5.536 | 1.225 | 10.148 | 41.790 | 2.469 | 0.43NS | **3.34***** |
| Propanedioic acid | 0.060 | 0.007 | 1.123NS | 0.067 | 0.011 | 9.781 | 0.583 | 0.059 | 0.16NS | **3.28**** |
| 3-Hydroxyisovaleric | 0.033 | 0.006 | 0.802NS | 0.026 | 0.003 | 4.514 | 0.147 | **0.014** | -0.34NS | **2.16**** |
| Butanedioic acid | 8.369 | 1.900 | 2.110 | 17.655 | 1.905 | 1.956 | 16.366 | 3.188 | 1.08* | 0.97ns |
| Malic acid | 4.490 | 0.263 | 2.285 | 10.262 | 0.652 | 2.407 | 10.807 | 1.426 | 1.19** | 1.27* |
| α-Ketoglutaric acid | 0.335 | 0.008 | 1.301NS | 0.436 | 0.064 | 2.097 | 0.702 | 0.040 | 0.38NS | 1.07** |
| L-(+)-Tartaric acid | 0.020 | 0.004 | 1.622 | 0.032 | 0.004 | 9.977 | 0.197 | 0.028 | 0.68ns | **3.30**** |
| 2-Aminoadipic acid | 0.076 | 0.016 | 2.094 | 0.159 | 0.024 | 5.133 | 0.389 | 0.066 | 1.06ns | **2.36*** |
| 3-phosphoglycerate | 0.089 | 0.025 | 2.052 | 0.182 | 0.043 | 0.175 | 0.015 | 0.003 | 1.03ns | -2.57ns |
| Glucaric acid | 0.162 | 0.024 | 0.826NS | 0.134 | 0.023 | 4.087 | 0.663 | **0.075** | -0.27NS | **2.03**** |
| D-Gluconic acid | 1.558 | 0.218 | 1.422NS | 2.215 | 0.407 | 14.408 | 22.443 | 2.101 | 0.51NS | **3.85**** |
| Galactaric acid | 1.566 | 0.187 | 1.519 | 2.379 | 0.200 | 2.661 | 4.166 | 0.897 | 0.60ns | 1.41ns |
| **Amino Acids** |  |  |  |  |  |  |  |  |  |  |
| L-Norleucine | 0.220 | 0.052 | 2.497 | 0.550 | 0.082 | 0.449 | 0.099 | 0.032 | 1.32* | -1.15ns |
| L-Alanine | 11.191 | 1.055 | 2.531 | 28.324 | 2.158 | 4.458 | 49.893 | 9.560 | 1.34** | **2.16*** |
| L-Threonine | 1.540 | 0.251 | 3.090 | 4.759 | 0.408 | 4.091 | 6.301 | 0.438 | 1.63** | **2.03**** |
| L-Asparagine | 0.635 | 0.092 | 3.186 | 2.024 | 0.314 | 0.258 | 0.164 | 0.016 | 1.67* | -1.95* |
| DL-Ornithine | 0.371 | 0.024 | 1.136NS | 0.421 | 0.044 | 2.579 | 0.956 | 0.164 | 0.18NS | 1.37** |
| L-Glutamine | 0.078 | 0.021 | 0.757NS | 0.059 | 0.007 | 117.900 | 9.167 | **1.274** | -0.40NS | **6.88**** |
| Tyramine | 0.317 | 0.028 | 0.776 | 0.246 | 0.011 | 2.269 | 0.720 | **0.074** | -0.37ns | 1.18* |
| L-Tyrosine | 0.130 | 0.030 | 1.844 | 0.240 | 0.051 | 2.633 | 0.343 | **0.029** | 0.88ns | 1.40* |
| Pyrrole-2-carboxylic acid | 0.107 | 0.023 | 0.740NS | 0.080 | 0.008 | 2.105 | 0.226 | **0.011** | -0.42NS | 1.08** |
| **Sugars** |  |  |  |  |  |  |  |  |  |  |
| D-Fructose | 25.044 | 0.919 | 2.009 | 50.307 | 6.484 | 3.913 | 98.003 | 7.439 | 1.01* | 1.97** |
| D-Mannose | 0.318 | 0.031 | 1.579 | 0.503 | 0.056 | 7.231 | 2.301 | 0.322 | 0.66ns | **2.86*** |
| D-Glucose | 21.228 | 2.976 | 1.422 | 30.179 | 3.292 | 2.910 | 61.764 | 10.381 | 0.51ns | 1.54* |
| Melibiose | 4.129 | 0.465 | 2.231 | 9.213 | 1.320 | 1.617 | 6.675 | 1.803 | 1.16* | 0.69NS |
| Sucrose | 0.075 | 0.011 | 0.554 | 0.041 | 0.004 | 3.321 | 0.247 | **0.037** | -0.87* | 1.72** |
| Maltose | 3.430 | 0.271 | 1.235NS | 4.237 | 0.437 | 2.342 | 8.033 | **0.887** | 0.30NS | 1.23* |
| Β- Gentiobiose | 0.631 | 0.114 | 1.882 | 1.187 | 0.221 | 7.115 | 4.487 | **0.393** | 0.91ns | **2.83**** |
| **Sugar Alcohols** |  |  |  |  |  |  |  |  |  |  |
| 1,3-Butanediol | 0.030 | 0.004 | 2.432 | 0.073 | 0.005 | 2.226NS | 0.067 | 0.008 | 1.28** | 1.16* |
| D-Pinitol | 2.045 | 0.200 | 1.269NS | 2.595 | 0.456 | 2.038 | 4.168 | 0.418 | 0.34NS | 1.03* |
| **Amines** | – |  |  | – |  |  | – |  | – | - |
| **Lipids and Sterols** |  |  |  |  |  |  |  |  |  |  |
| 1-Monolinolein | 0.037 | 0.006 | 6.997 | 0.257 | 0.030 | 9.851 | 0.361 | 0.055 | **2.80**** | **3.29**** |
| Stigmasterol | 0.122 | 0.004 | 2.320 | 0.282 | 0.022 | 2.427 | 0.295 | 0.030 | 1.21** | 1.27* |

Folds changes were using the formula log_2_ ^(treatment/control)^
