# Supplementary material for: Morphophysiological and Comparative Metabolic Profiling of Purslane Genotypes (Portulaca oleracea L.) under Salt Stress
Source: Biomed Res Int. 2020 Jun 17;2020:4827045. doi: 10.1155/2020/4827045 (PMC7321505; doi:10.1155/2020/4827045)
Supplement: Supplementary Materials — Supplementary Figure S1: (A) “Tall Green” local (“TG”—American origin), (B) a wild variety “Shandong, China” local (“SD”). Supplementary Table S1: metabolites detected by GC-MS from “TG” and “SD” leaves of purslane cultivars at 0, 100, and 200 mM salinity stress. Supplementary Table S2: metabolites detected by GC-MS from “TG” and “SD” roots of purslane cultivars at 0, 100, and 200 mm salinity stress. Supplementary Table S3: Shandong Wild leaves and roots for fold change. Supplementary Table S4: Tall Green leaves and roots for fold change. [file 4827045.f1.zip › 4827045.f1/TG- Tall Green Pathway.docx]

Glucose

Sucrose

Legend:

100 m M/Ck

200 m M/Ck

P-value:

< 0.05

< 0.01

D-Gluconate

D-Fructose

Nurleucine

Shikimic acid

PEP

Mannose

G6P

Maltose

Myo-inositol

F6P

L

L-Serine

L

3PGA

Quinate

Tyrosine

Phenylalanine

Aspargin

Pyruvate

L

Lysine

L-Alanin

Tryptophan

Aspartate

Acetyl -CoA

L

Palmitic acid

Oleic acid

Linolenic acid

Threonine

L

Citrate

Oxalic acid

Cis-Aconitic acid

L

Glutamine

Nicotinate

L

L

D-Isocitrate

TCA Cycle

Succinate

1. Ketoglutarate

Malate

Fumarate

DL-Ornithine

2-Butenedioic acid

Glutamic acid

Cadaverine

Piperdine
