# Supplementary figures and images for: Morphophysiological and Comparative Metabolic Profiling of Purslane Genotypes (Portulaca oleracea L.) under Salt Stress
Source: Biomed Res Int. 2020 Jun 17;2020:4827045. doi: 10.1155/2020/4827045 (PMC7321505; doi:10.1155/2020/4827045)

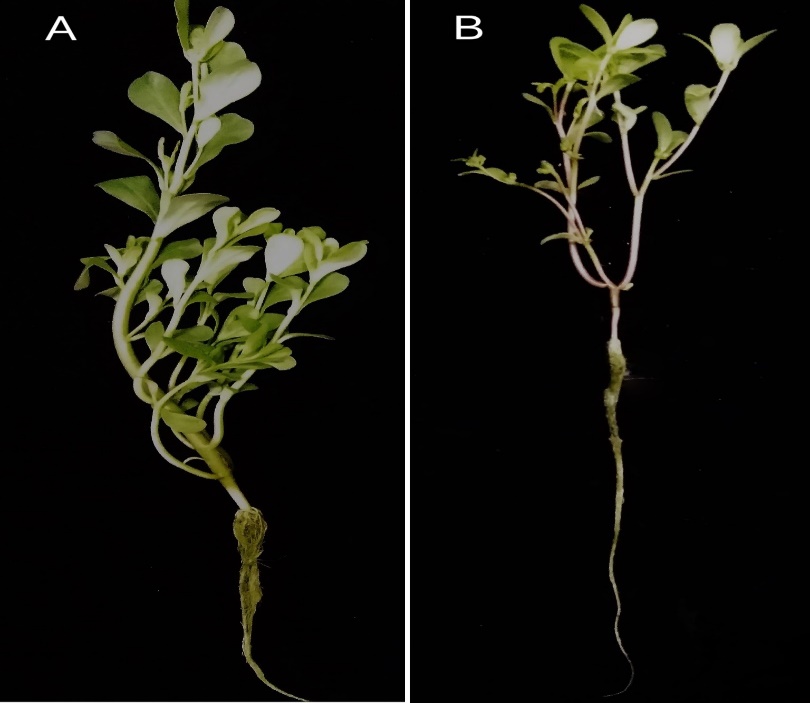


Figure S1 A: “Tall Green” local (‘TG’- American origin), B: a wild variety “Shandong, China” local (‘SD’)

Supplement: Supplementary Materials — Supplementary Figure S1: (A) “Tall Green” local (“TG”—American origin), (B) a wild variety “Shandong, China” local (“SD”). Supplementary Table S1: metabolites detected by GC-MS from “TG” and “SD” leaves of purslane cultivars at 0, 100, and 200 mM salinity stress. Supplementary Table S2: metabolites detected by GC-MS from “TG” and “SD” roots of purslane cultivars at 0, 100, and 200 mm salinity stress. Supplementary Table S3: Shandong Wild leaves and roots for fold change. Supplementary Table S4: Tall Green leaves and roots for fold change. [file 4827045.f1.zip › 4827045.f1/Figure S1.docx]

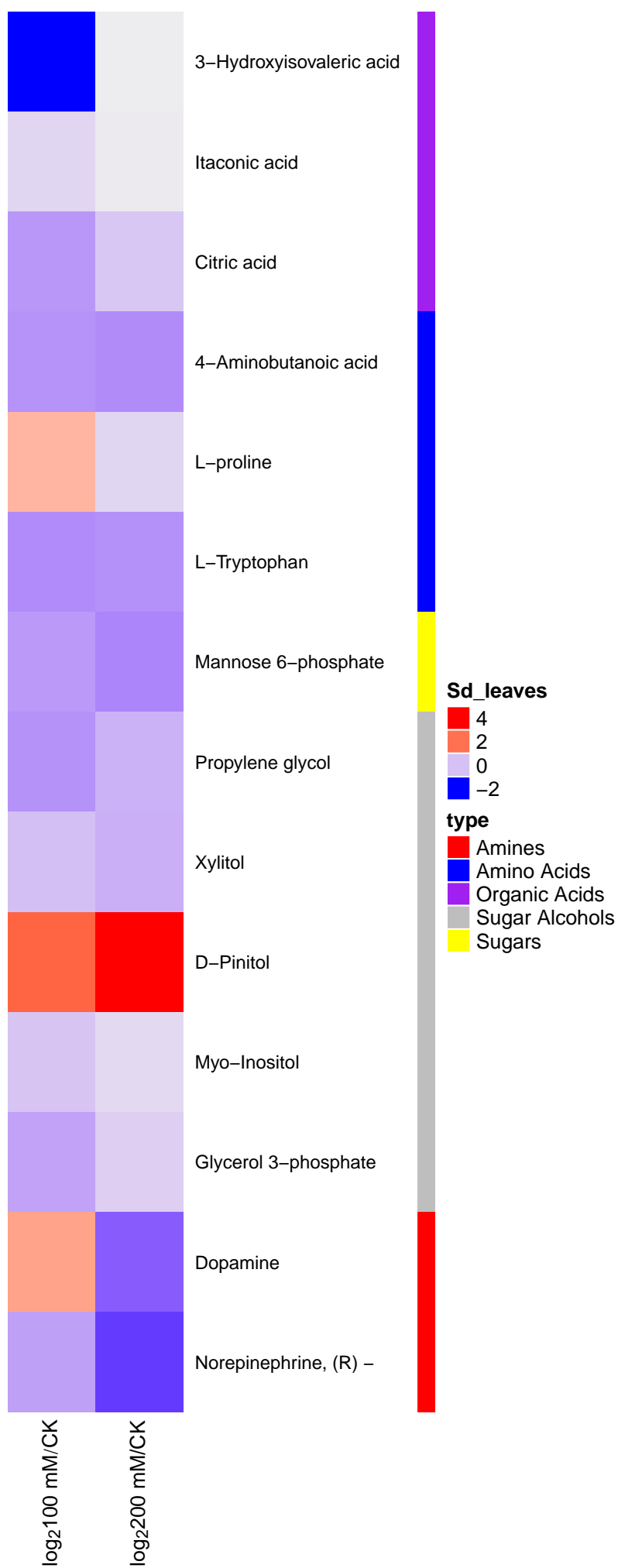

Supplement: Supplementary Materials — Supplementary Figure S1: (A) “Tall Green” local (“TG”—American origin), (B) a wild variety “Shandong, China” local (“SD”). Supplementary Table S1: metabolites detected by GC-MS from “TG” and “SD” leaves of purslane cultivars at 0, 100, and 200 mM salinity stress. Supplementary Table S2: metabolites detected by GC-MS from “TG” and “SD” roots of purslane cultivars at 0, 100, and 200 mm salinity stress. Supplementary Table S3: Shandong Wild leaves and roots for fold change. Supplementary Table S4: Tall Green leaves and roots for fold change. [file 4827045.f1.zip › 4827045.f1/SD-leave.pdf]

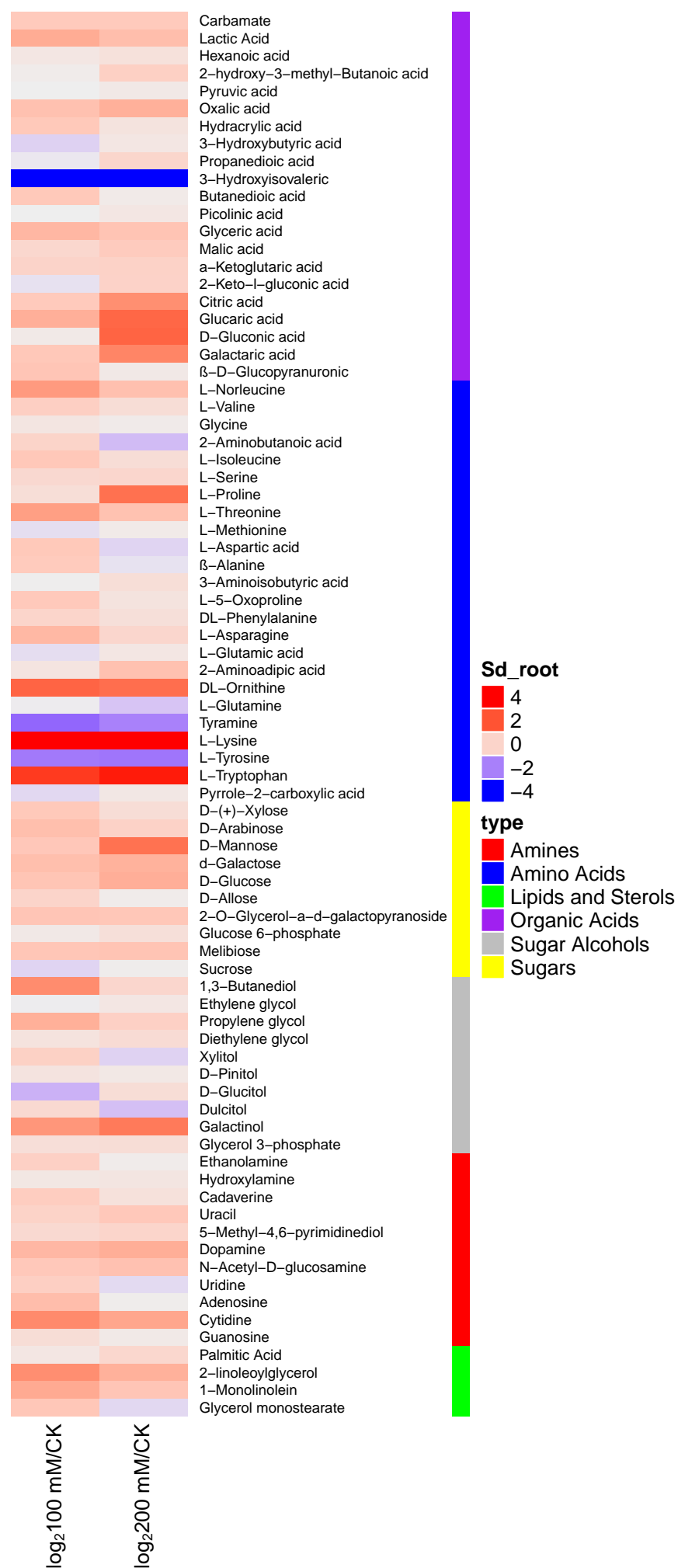

Supplement: Supplementary Materials — Supplementary Figure S1: (A) “Tall Green” local (“TG”—American origin), (B) a wild variety “Shandong, China” local (“SD”). Supplementary Table S1: metabolites detected by GC-MS from “TG” and “SD” leaves of purslane cultivars at 0, 100, and 200 mM salinity stress. Supplementary Table S2: metabolites detected by GC-MS from “TG” and “SD” roots of purslane cultivars at 0, 100, and 200 mm salinity stress. Supplementary Table S3: Shandong Wild leaves and roots for fold change. Supplementary Table S4: Tall Green leaves and roots for fold change. [file 4827045.f1.zip › 4827045.f1/SD-Roots.pdf]

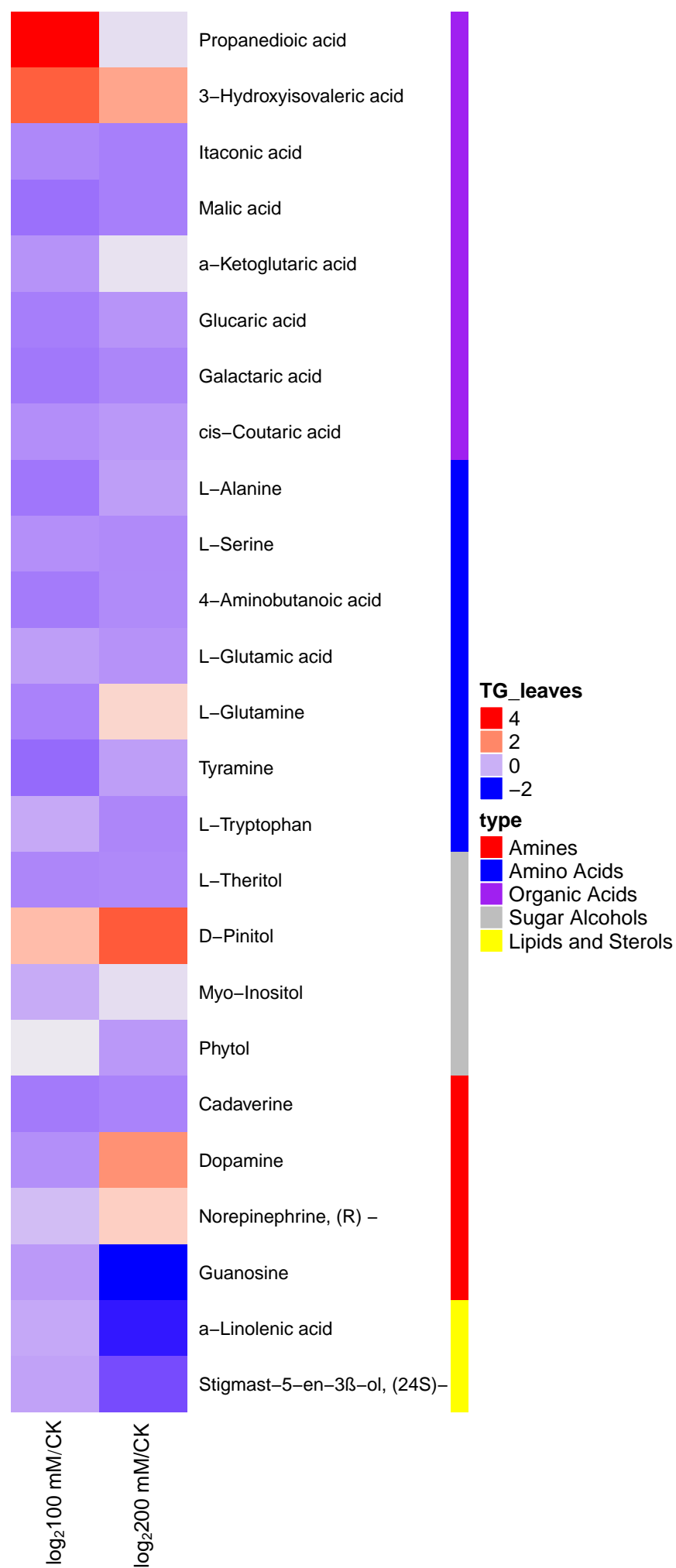

Supplement: Supplementary Materials — Supplementary Figure S1: (A) “Tall Green” local (“TG”—American origin), (B) a wild variety “Shandong, China” local (“SD”). Supplementary Table S1: metabolites detected by GC-MS from “TG” and “SD” leaves of purslane cultivars at 0, 100, and 200 mM salinity stress. Supplementary Table S2: metabolites detected by GC-MS from “TG” and “SD” roots of purslane cultivars at 0, 100, and 200 mm salinity stress. Supplementary Table S3: Shandong Wild leaves and roots for fold change. Supplementary Table S4: Tall Green leaves and roots for fold change. [file 4827045.f1.zip › 4827045.f1/TG-leave.pdf]

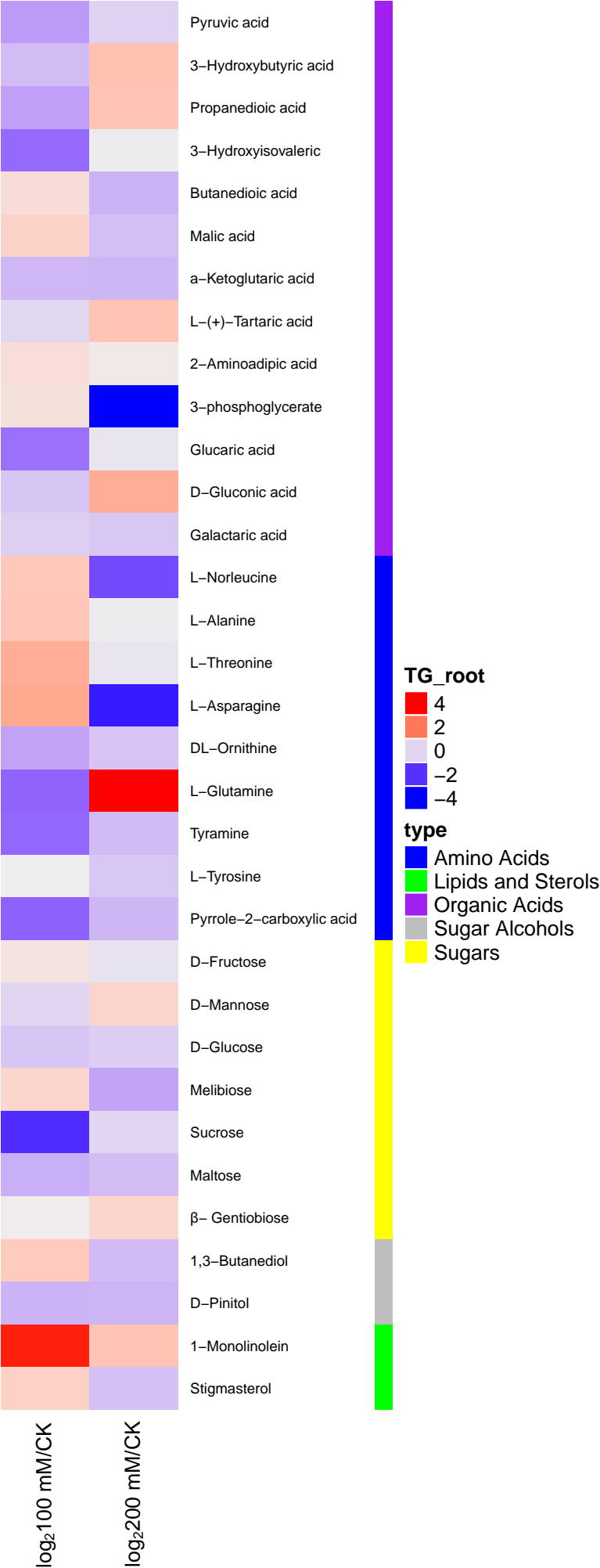

Supplement: Supplementary Materials — Supplementary Figure S1: (A) “Tall Green” local (“TG”—American origin), (B) a wild variety “Shandong, China” local (“SD”). Supplementary Table S1: metabolites detected by GC-MS from “TG” and “SD” leaves of purslane cultivars at 0, 100, and 200 mM salinity stress. Supplementary Table S2: metabolites detected by GC-MS from “TG” and “SD” roots of purslane cultivars at 0, 100, and 200 mm salinity stress. Supplementary Table S3: Shandong Wild leaves and roots for fold change. Supplementary Table S4: Tall Green leaves and roots for fold change. [file 4827045.f1.zip › 4827045.f1/TG-roots.pdf]
