# Supplementary material for: Morphophysiological and Comparative Metabolic Profiling of Purslane Genotypes (Portulaca oleracea L.) under Salt Stress
Source: Biomed Res Int. 2020 Jun 17;2020:4827045. doi: 10.1155/2020/4827045 (PMC7321505; doi:10.1155/2020/4827045)
Supplement: Supplementary Materials — Supplementary Figure S1: (A) “Tall Green” local (“TG”—American origin), (B) a wild variety “Shandong, China” local (“SD”). Supplementary Table S1: metabolites detected by GC-MS from “TG” and “SD” leaves of purslane cultivars at 0, 100, and 200 mM salinity stress. Supplementary Table S2: metabolites detected by GC-MS from “TG” and “SD” roots of purslane cultivars at 0, 100, and 200 mm salinity stress. Supplementary Table S3: Shandong Wild leaves and roots for fold change. Supplementary Table S4: Tall Green leaves and roots for fold change. [file 4827045.f1.zip › 4827045.f1/Metabolomic Table S2-roots.doc]

Table S2. Metabolites detected by GC-MS from ‘TG’ and ‘SD’ roots of purslane cultivars at 0, 100 mm and 200 mm salinity stress.

| **Metabolites** | **‘TG’** | | | | |  | **‘SD’** | | |  |
| --- | --- | --- | --- | --- | --- | --- | --- | --- | --- | --- |
|  | 0 mM |  | 100 mM |  | 200mM | 0 mM | 100mM | | | 200 mM |
| **Organic Acids** | Average SE | C% | Average SE | C% | Average SE | Average SE | C% | Average SE | C% | Average SE |
| Carbamate | 0.755± 0.017 a | 1.139 | 0.860 ±0.119 b | 1.282 | 0.967± 0.105 a | 0.594± 0.052 c | 2.884 | 1.714±0.211 b | 4.344 | 2.582±0.211a |
| Lactic Acid | 17.921± 1.892 a | 0.986 | 17.668± 2.105 a | 0.979 | 17.548± 2.014 a | 0.594±0.056 c | 5.184 | 3.082±0.262 b | 5.449 | 3.239±0.472a |
| Hexanoic acid | 0.067± 0.007 b | 0.940 | 0.063± 0.005 b | 1.303 | 0.087± 0.009 a | 0.017±0.002 b | 1.631 | 0.028±0.005 a | 2.693 | 0.047±0.006a |
| Glycolic acid | 0.395± 0.004 a | 1.007 | 0.398± 0.098 a | 0.964 | 0.381± 0.036 b | 0.107±0.009 b | 2.534 | 0.272±0.033 b | 2.826 | 0.303±0.041a |
| 2-hydroxy-3-methyl-Butanoic acid | ND | ND | ND | ND | ND | 0.014±0.002 b | 1.503 | 0.021±0.002 a | 3.818 | 0.054±0.005a |
| Pyruvic acid | 1.249± 0.061 b | 1.089 | 1.360± 0.256 b | 3.086 | 3.855± 0.354 a | 0.594±0.024 b | 1.410 | 0.838±0.050 b | 2.369 | 1.408±0.191a |
| Oxalic acid | 37.642± 2.867 a | 1.094 | 41.182 ±1.429 a | 0.193 | 7.283± 0.532 b | 4.011±0.162 c | 3.458 | 13.871±3.340 b | 7.223 | 28.975±1.732a |
| Hydracrylic acid | 0.549± 0.050 a | 0.565 | 0.310 ±0.024 b | 0.546 | 0.300± 0.032 b | 0.062±0.011 b | 2.967 | 0.184±0.005 a | 2.647 | 0.164±0.021a |
| 3-Hydroxybutyric acid | 4.118± 0.595 b | 1.344 | 5.536± 1.225 b | 10.148 | 41.790± 2.469 a | 3.638±0.131 b | 0.896 | 3.261±0.283 b | 2.490 | 9.059±1.157a |
| Propanedioic acid | 0.060± 0.007 b | 1.123 | 0.067± 0.011 b | 9.781 | 0.583± 0.059 a | 0.026±0.004 b | 1.252 | 0.033±0.004 b | 3.378 | 0.089±0.004a |
| 3-Hydroxyisovaleric | 0.033± 0.006 b | 0.802 | 0.026± 0.003 b | 4.514 | 0.147± 0.014 a | 3.638±0.002 b | 0.641 | 0.010±0.002 b | 2.557 | 0.042±0.007a |
| α-Ketoisocaproic acid | 0.671± 0.216 a | 0.377 | 0.253± 0.041 b | 0.782 | 0.525± 0.053 a | 0.157±0.012 a | 1.028 | 0.161±0.021 a | 1.086 | 0.170±0.009a |
| Benzoic Acid | 1.147± 0.196 b | 0.291 | 0.334± 0.023 b | 0.309 | 0.355± 0.039 a | 1.082±0.091 a | 0.118 | 0.127±0.009 b | 0.266 | 0.287±0.033b |
| Butanedioic acid | 8.369± 1.900 b | 2.110 | 17.655± 1.905 a | 1.956 | 16.366± 3.188 a | 3.402±0.273 b | 2.962 | 10.077±2.268 a | 2.308 | 7.852±0.202a |
| Picolinic acid | 0.108± 0.020 b | 0.620 | 0.067± 0.003 b | 7.306 | 0.791± 0.156 a | 0.041±0.008 b | 1.410 | 0.058±0.003 b | 2.508 | 0.102±0.014a |
| Glyceric acid | 1.793± 0.150 a | 1.019 | 1.827± 0.372 a | 0.803 | 1.439± 0.145 a | 0.179±0.016 b | 4.205 | 0.753±0.070 a | 4.879 | 0.874±0.087a |
| 2-Butenedioic acid | 0.296± 0.028 a | 0.968 | 0.287± 0.039 a | 0.616 | 0.183± 0.021 b | 0.096±0.008 b | 3.464 | 0.331±0.021 a | 0.991 | 0.095±0.012b |
| Pentanedioic acid | 0.424± 0.103 a | 0.959 | 0.407± 0.020 a | 0.397 | 0.168± 0.030 b | 0.090±0.005 b | 1.744 | 0.156±0.031 a | 1.055 | 0.094±0.011a |
| Malic acid | 4.490± 0.263 b | 2.285 | 10.262± 0.652 a | 2.407 | 10.807± 1.426 a | 1.973±0.315 c | 2.215 | 4.370±0.509 b | 4.236 | 8.359±0.156a |
| L-Threonic acid | 0.280± 0.047 a | 0.768 | 0.215± 0.039 a | 0.764 | 0.214± 0.030 a | 0.105±0.016 b | 0.629 | 0.066±0.007 b | 2.049 | 0.216±0.040a |
| α-Ketoglutaric acid | 0.335± 0.008 b | 1.301 | 0.436± 0.064 b | 2.097 | 0.702± 0.040 a | 0.088±0.012 c | 2.410 | 0.211±0.011 b | 3.732 | 0.327±0.020a |
| L-(+)-Tartaric acid | 0.020± 0.004 b | 1.622 | 0.032± 0.004 b | 9.977 | 0.197± 0.028 a | 0.000±0.000 b | 1.000 | 0.000±0.000 b | ------- | 0.056±0.004a |
| 2-Aminoadipic acid | 0.076± 0.016 b | 2.094 | 0.159± 0.024 b | 5.133 | 0.389± 0.066 a | ND | ND | ND | ND | ND |
| 2-Keto-l-gluconic acid | 0.130± 0.011 b | 0.954 | 0.124±0.030 b | 1.286 | 0.167± 0.025 a | 0.033±0.005 b | 1.172 | 0.038±0.004 b | 3.703 | 0.121±0.004a |
| 3-phosphoglycerate | 0.089± 0.025 a | 2.052 | 0.182± 0.043 a | 0.175 | 0.015±0.003 a | 0.016±0.002 a | 0.098 | 0.015±0.002 a | 1.069 | 0.017±0.003a |
| Citric acid | 2.698±0.161 b | 1.469 | 3.963±0.526 a | 1.524 | 4.110±0.691 b | 0.965±0.124 b | 2.903 | 2.800±0.554 b | 13.347 | 12.874±0.820a |
| Quininic acid | 0.172±0.019 a | 1.300 | 0.224±0.026 a | 0.312 | 0.054±0.004 a | 0.169±0.023 a | 1.065 | 0.180±0.028 a | 1.265 | 0.214±0.025a |
| Glucaric acid | 0.162±0.024 a | 0.826 | 0.134±0.023 a | 4.087 | 0.663±0.075 b | 0.019±0.003 c | 4.860 | 0.092±0.014 b | 26.097 | 0.495±0.015a |
| Pantothenic acid | 0.140±0.018 b | 1.048 | 0.146±0.014 b | 0.352 | 0.049±0.001 a | 0.045±0.004 c | 1.311 | 0.059±0.008 b | 0.566 | 0.025±0.002a |
| D-Gluconic acid | 1.558±0.218 a | 1.422 | 2.215±0.407 a | 14.408 | 22.443±2.101 b | 0.293±0.014 a | 1.554 | 0.456±0.018 a | 27.547 | 8.073±0.445b |
| Galactaric acid | 1.566±0.187 b | 1.519 | 2.379±0.200 b | 2.661 | 4.166±0.897 a | 0.120±0.015 b | 3.023 | 0.363±0.023 b | 15.915 | 1.911±0.105a |
| β-D-Glucopyranuronic | 2.853±0.067 a | 1.148 | 3.274±0.167 a | 0.478 | 1.362± 0.177 a | 0.779±0.072 c | 3.150 | 2.454±0.150 a | 2.370 | 1.846±0.172b |

| **Metabolites** |  | **‘TG’** | | | | | |  | **‘SD’** | | |  |
| --- | --- | --- | --- | --- | --- | --- | --- | --- | --- | --- | --- | --- |
|  | 0 mM | |  | 100 mM |  | 200mM | 0 mM |  | 100 mM 200 mM | | | |
| **Amino acids** | Average SE | | C% | Average SE | C% | Average SE | Average SE | C% | Average SE | C% | Average SE | |
| L-Norleucine | 0.220± 0.052 b | | 2.497 | 0.550 ±0.082 a | 0.449 | 0.099± 0.032 b | 0.066±0.006 b | 7.164 | 0.475± 0.075 a | 5.270 | 0.349± 0.044 a | |
| L-Valine | 26.838 ±1.819 b | | 1.176 | 31.571± 2.677 a | 1.327 | 35.622± 2.397 a | 4.369±0.447 b | 2.600 | 11.360± 0.909 a | 2.959 | 12.930±0.782 a | |
| L-Alanine | 11.191± 1.055 b | | 2.531 | 28.324± 2.158 b | 4.458 | 49.893± 9.560 a | 13.195±0.375 a | 0.851 | 11.226± 0.892 b | 1.031 | 13.602±0.690 a | |
| Glycine | 18.039± 2.772 a | | 0.928 | 16.735± 1.233 a | 1.267 | 22.859± 1.442 a | 2.703± 0.201 b | 1.701 | 4.599± 0.787 b | 2.270 | 6.136± 0.294 a | |
| L-Leucine | 3.673 ±0.341 a | | 0.655 | 2.405± 0.349 b | 1.148 | 4.216± 0.570 a | 5.452±1.290 a | 1.396 | 7.608± 1.009 a | 1.516 | 8.264± 0.392 a | |
| 2-Aminobutanoic acid | 0.388 ±0.037 a | | 1.091 | 0.423± 0.033 a | 0.487 | 0.189± 0.059 b | 0.075± 0.006 b | 2.381 | 0.179± 0.006 a | 0.929 | 0.070± 0.004 b | |
| L-Isoleucine | 14.785± 1.153 b | | 1.200 | 17.735± 2.233 a | 1.440 | 21.285± 1.955 a | 2.023±0.200 b | 3.017 | 6.104±0.477 a | 2.980 | 6.029± 0.277 a | |
| L-Serine | 12.592± 2.040 b | | 1.212 | 15.255± 1.122 b | 1.687 | 21.243± 0.458 a | 2.520± 0.243 c | 2.174 | 5.480±0.933 b | 3.406 | 8.585±0.676 a | |
| L-Proline | 4.175 ±0.771 b | | 1.193 | 4.979± 0.098 b | 7.859 | 32.810±6.298 a | 1.907± 0.507 b | 1.945 | 3.708±0.307 b | 22.425 | 42.757± 7.609 a | |
| .L-Threonine | 1.540 ±0.251 c | | 3.090 | 4.759± 0.408 b | 4.091 | 6.301± 0.438 a | 0.239±0.017 c | 6.648 | 1.588±0.104 a | 5.105 | 1.220±0.154 b | |
| L-Methionine | 1.433 ±0.138 a | | 0.719 | 1.031± 0.176 a | 0.313 | 0.448± 0.070 b | 0.226±0.021 b | 1.092 | 0.247±0.035 b | 2.306 | 0.522± 0.040 a | |
| L-Aspartic acid | 17.212± 1.429 b | | 1.176 | 20.246± 1.255 b | 1.741 | 29.964± 5.690 a | 5.283±0.454 b | 2.958 | 15.629±0.994 a | 1.398 | 7.388± 0.567 b | |
| β-Alanine | 4.656 ±0.603 b | | 1.555 | 7.241± 0.595 a | 0.603 | 2.808± 0.582 b | 0.395±0.046 c | 2.831 | 1.119±0.101 a | 1.734 | 0.686± 0.077 b | |
| 3-Aminoisobutyric acid | 0.281 ±0.010 a | | 1.069 | 0.301± 0.022 a | 0.706 | 0.199± 0.026 b | 0.044±0.005 b | 1.443 | 0.063±0.009 b | 2.956 | 0.129±0.014 a | |
| L-5-Oxoproline | 40.015 ±4.442 b | | 1.496 | 59.860± 7.820 a | 0.842 | 33.693± 4.091 b | 8.475±0.671 b | 2.926 | 24.795±2.954 a | 2.644 | 22.407± 2.947 a | |
| 4-Aminobutanoic acid | 55.185 ±3.943 b | | 1.780 | 98.241± 2.372 a | 1.664 | 91.820±12.385 a | 0.797± 0.099 b | 1.896 | 1.510±0.222 a | 1.608 | 1.281± 0.162 a | |
| DL-Phenylalanine | 7.187 ±0.823 a | | 0.757 | 5.442± 0.521 a | 0.845 | 6.073± 0.526 a | 0.975± 0.110 b | 2.315 | 2.258±0.231 a | 2.859 | 2.788±0.207 a | |
| L-Asparagine | 0.635 ±0.092 b | | 3.186 | 2.024± 0.314 a | 0.258 | 0.164± 0.016 b | 0.098± 0.016 b | 4.130 | 0.405±0.092 a | 3.381 | 0.332± 0.026 a | |
| L-Glutamic acid | 19.143 ±2.625 b | | 1.031 | 19.730± 2.103 b | 1.996 | 38.215± 3.171 a | 5.050± 0.339 b | 1.070 | 5.403±0.498 b | 2.475 | 12.497± 1.899 a | |
| 2-Aminoadipic acid | ND | | ND | ND | ND | ND | 0.022±0.003 b | 1.758 | 0.038±0.007 b | 5.300 | 0.114±0.007 a | |
| DL-Ornithine | 0.371 ±0.024 b | | 1.136 | 0.421± 0.044 b | 2.579 | 0.956± 0.164 a | 0.245±0.004 b | 1.816 | 4.422±0.929 a | 17.310 | 5.700± 0.144 a | |
| L-Glutamine | 0.078 ±0.021 b | | 0.757 | 0.059± 0.007 b | 117.900 | 9.167± 1.274 a | 0.329±0.167 b | 13.428 | 0.444±0.069 a | 1.454 | 0.356± 0.039 a | |
| Tyramine | 0.317 ±0.028 b | | 0.776 | 0.246± 0.011 b | 2.269 | 0.720± 0.074 a | 0.245±0.004 b | 1.237 | 0.039±0.006 b | 2.855 | 0.090±0.008 a | |
| L-Lysine | 4.183 ±0.389 b | | 0.078 | 0.327± 0.017 c | 1.605 | 6.712± 1.014 a | 0.032±0.008 c | 3.180 | 1.568±0.084 b | 4.821 | 2.377±0.083 a | |
| L-Tyrosine | 0.130± 0.030 b | | 1.844 | 0.240± 0.051 a | 2.633 | 0.343±0.029 a | 0.493± 0.042 c | 2.041 | 0.106±0.015 b | 2.889 | 0.150±0.005 a | |
| L-Tryptophan | 6.978± 0.423 a | | 0.272 | 1.899±0.392 b | 1.083 | 7.556±0.466 a | 0.052± 0.005 c | 1.734 | 1.623±0.252 b | 3.307 | 3.094±0.246 a | |
| Pyrrole-2-carboxylic acid | 0.107± 0.023 b | | 0.740 | 0.080± 0.008 b | 2.105 | 0.226± 0.011 a | 0.020±0.002b | 2.463 | 0.049± 0.003b | 13.448 | 0.270±0.049a | |

| **Metabolites** |  | **‘TG’** | | | | |  | | **‘SD’** | |  |
| --- | --- | --- | --- | --- | --- | --- | --- | --- | --- | --- | --- |
|  | 0 mM | |  | 100 mM |  | 200mM | 0 mM |  | 100 mM | | 200 mM |
| **Sugars** | Average SE | | C% | Average SE | C% | Average SE |  | C% | Average SE | C% | Average SE |
| D-(+)-Xylose | 0.762± 0.035 b | | 1.503 | 1.146± 0.183 a | 0.789 | 0.602±0.032 b | 0.185±0.022 b | 2.958 | 0.549±0.052 a | 2.981 | 0.553±0.063 a |
| D-Arabinose | 5.733± 0.522 a | | 0.939 | 5.386± 0.507 a | 1.056 | 6.051±0.839 a | 0.677±0.076 b | 3.573 | 2.420±0.227 a | 3.679 | 2.492± 0.157 a |
| D-Fructose | 25.044±0.919 c | | 2.009 | 50.307±6.484 b | 3.913 | 98.003±7.439 a | 11.740±0.955 c | 2.524 | 29.627±1.718 b | 5.508 | 64.662±5.145a |
| Fructose 6-phosphate | 0.402± 0.048 b | | 1.218 | 0.490± 0.080 b | 1.849 | 0.744±0.054 a | 0.128±0.007 b | 1.565 | 0.201±0.010 a | 1.320 | 0.169±0.014 a |
| D-Mannose | 0.318± 0.031 b | | 1.579 | 0.503± 0.056 b | 7.231 | 2.301±0.322 a | 0.063±0.008 b | 3.028 | 0.191±0.032 b | 22.090 | 1.393±0.076 a |
| Mannose 6-phosphate | 0.943± 0.098 a | | 1.141 | 1.076± 0.197 a | 1.244 | 1.173± 0.316 a | 0.356± 0.040 a | 0.978 | 0.349±0.077 a | 1.001 | 0.357±0.040 a |
| d-Galactose | 1.393± 0.045 a | | 1.257 | 1.751± 0.251 a | 0.677 | 0.944± 0.039 b | 0.172± 0.025 c | 3.605 | 0.618±0.087 b | 6.964 | 1.195±0.059 a |
| D-Glucose | 21.228± 2.976 b | | 1.422 | 30.179± 3.292 b | 2.910 | 61.764±10.381 a | 7.449± 0.678 c | 3.198 | 23.821±1.526 b | 7.428 | 55.332± 6.824a |
| D-Allose | 0.532± 0.031 a | | 0.545 | 0.290± 0.091 b | 0.403 | 0.214± 0.028 b | 0.102± 0.026 a | 2.348 | 0.241±0.076 a | 2.235 | 0.229±0.021 a |
| 2-O-Glycerol-α-d-galactopyranoside | 6.448± 1.238 a | | 0.598 | 3.858± 0.804 a | 0.947 | 6.105±0.724 a | 0.942± 0.076 c | 3.173 | 2.988±0.384 b | 4.587 | 4.319± 0.295a |
| Glucose 6-phosphate | 0.678± 0.045 a | | 1.082 | 0.733± 0.064 a | 0.473 | 0.321±0.034 b | 0.084± 0.007 c | 1.583 | 0.133±0.017 b | 2.864 | 0.241± 0.017a |
| D-Lactose | 3.644± 0.219 a | | 0.917 | 3.343± 0.432 a | 0.272 | 0.991±0.181 b | 2.001± 0.426 a | 1.345 | 2.691±0.715 a | 0.769 | 1.538±0.181 a |
| Maltose | 3.430± 0.271 b | | 1.235 | 4.237± 0.437 b | 2.342 | 8.033±0.887 a | 2.061± 0.148 b | 1.531 | 3.155±0.556 b | 2.792 | 5.755± 0.441a |
| β-Gentiobiose | 0.631± 0.114 b | | 1.882 | 1.187± 0.221 b | 7.115 | 4.487±0.393 a | 0.763± 0.055 b | 0.207 | 0.158±0.033 a | 1.044 | 0.796± 0.038a |
| D-Trehalose | 0.326± 0.051 a | | 0.576 | 0.188± 0.035 b | 0.521 | 0.170±0.033 b | ND | ND | ND | ND | ND |
| Melibiose | 4.129± 0.465 b | | 2.231 | 9.213± 1.320 a | 1.617 | 6.675±1.803 a | 1.112±0.178 c | 3.111 | 3.460±0.535 b | 4.888 | 5.435± 0.895a |
| Sucrose | 0.075± 0.011 b | | 0.554 | 0.041± 0.004 b | 3.321 | 0.247±0.037 a | 0.112±0.009 b | 0.940 | 0.105± 0.021 b | 2.195 | 0.246±0.012 a |
| **Sugar alchols** |  | |  |  |  |  |  |  |  |  |  |
| 1,3-Butanediol | 0.030± 0.004 b | | 2.432 | 0.073±0.005 a | 2.226 | 0.067±0.008 a | 0.018±0.003 b | 9.012 | 0.167±0.023 b | 3.283 | 0.061±0.011a |
| Ethylene glycol | 7.475± 0.405 a | | 0.893 | 6.679±1.110 a | 1.095 | 8.184±1.167 a | 8.149±0.579 b | 1.368 | 11.147±1.044 a | 2.466 | 20.097± 1.716a |
| Propylene glycol | 0.139±0.008 b | | 0.867 | 0.121±0.017 b | 1.758 | 0.245±0.044 a | 0.038±0.005 b | 4.889 | 0.183±0.031 a | 3.870 | 0.145±0.020 b |
| Diethylene glycol | 0.114±0.006 a | | 0.902 | 0.103 ±0.010 b | 1.649 | 0.188±0.042 a | 0.013±0.002 c | 1.755 | 0.023±0.002 b | 3.001 | 0.040±0.003 a |
| Glycerol | 14.472±0.293 b | | 0.844 | 12.217± 1.537 b | 1.532 | 22.168±1.127 a | 6.281±1.226 b | 1.299 | 8.161±1.165 b | 1.901 | 11.940±0.999 a |
| Xylitol | 0.486±0.017 a | | 0.822 | 0.400±0.043 a | 0.318 | 0.155±0.008 b | 0.061±0.007 b | 2.544 | 0.154±0.024 a | 1.364 | 0.083±0.007 b |
| D-Pinitol | 2.045±0.200 b | | 1.269 | 2.595±0.456 b | 2.038 | 4.168±0.418 a | 1.494±0.330 b | 1.749 | 2.613±0.272 a | 2.393 | 3.575± 0.350 a |
| D-Glucitol | 1.239± 0.137 a | | 0.587 | 0.727±0.087 b | 0.479 | 0.594±0.059 b | 0.279±0.063 b | 0.529 | 0.148±0.020 b | 2.989 | 0.835±0.034 a |
| Myo-Inositol | 13.136±2.714 a | | 0.970 | 12.737±2.355 a | 1.160 | 15.231±1.768 a | 9.279± 1.628 b | 1.302 | 12.082±1.542 a | 1.781 | 16.528±1.359 a |
| Inositol monophosphate | 1.683±0.303 a | | 1.211 | 2.039±0.120 a | 0.410 | 0.690±0.146 b | 0.356±0.024 a | 1.975 | 0.702±0.227 a | 1.212 | 0.431±0.044 a |
| Phytol | 0.143±0.008 a | | 1.376 | 0.197±0.028 a | 1.146 | 0.164±0.024 a | 0.042±0.007 a | 1.458 | 0.061±0.011 a | 1.577 | 0.066±0.008 a |
| Dulcitol | ND | | ND | ND | ND | ND | 0.016±0.001 b | 2.118 | 0.034±0.003 a | 0.974 | 0.016±0.003 b |
| Galactinol | 0.277±0.043 b | | 2.932 | 0.813±0.024 a | 0.906 | 0.251±0.046 b | 0.084±0.005 b | 7.735 | 0.648±0.080 a | 19.134 | 1.602± 0.062 b |
| d-Glycero-l-gluco-heptose | ND | | ND | ND | ND | ND | 0.300±0.035 c | 1.762 | 0.529±0.075 b | 0.823 | 0.247±0.022 a |
| Glycerol 3-phosphate | 6.525±0.488 a | | 0.851 | 5.554±0.801 a | 1.170 | 7.637±1.381 a | 1.194±0.103 c | 1.937 | 2.313± 0.260 b | 2.989 | 3.569± 0.065 a |

| **Metabolites** | **‘TG’** | | | | |  | **‘SD’** | | |  |
| --- | --- | --- | --- | --- | --- | --- | --- | --- | --- | --- |
|  | 0 mM | C% | 100 mM | C% | 200mM | 0 mM | 100 mM | | | 200 mM |
| **Amines** | Average SE |  | Average SE |  | Average SE | Average SE | C% | Average SE | C% | Average SE |
| Ethanolamine | 41.381± 5.623 a | 1.065 | 44.078±3.660 a | 0.880 | 36.428±1.675 a | 9.217±0.869 b | 2.547 | 23.470±3.24 a | 2.227 | 20.528±1.698 a |
| Hydroxylamine | 0.045± 0.002 a | 1.090 | 0.049±0.006 a | 1.180 | 0.053± 0.003 a | 0.011±0.001 b | 1.727 | 0.018±0.003 b | 2.614 | 0.028± 0.003 a |
| Cadaverine | 0.152± 0.006 b | 0.865 | 0.132±0.039 b | 2.510 | 0.383± 0.039 a | 0.365±0.035 b | 2.741 | 1.000±0.034 a | 2.768 | 1.010±0.035 a |
| Uracil | 1.971± 0.233 a | 0.649 | 1.279±0.237 b | 1.521 | 2.999± 0.450 a | 0.168±0.014 c | 2.429 | 0.408±0.083 b | 4.468 | 0.750± 0.036 a |
| 5-Methyl-4,6-pyrimidinediol | 0.186± 0.007 a | 0.481 | 0.090± 0.014 b | 0.997 | 0.186± 0.036 a | 0.015±0.002 c | 2.147 | 0.032±0.006 b | 3.486 | 0.052± 0.007 a |
| Ammelide | 0.103± 0.006 a | 0.439 | 0.045± 0.002 b | 0.892 | 0.092± 0.008 a | 0.049±0.008 a | 1.137 | 0.056±0.009 a | 0.352 | 0.017± 0.002 b |
| Putrescine | 2.638± 0.164 b | 1.570 | 4.143± 0.292 a | 1.366 | 3.602± 0.477 a | 3.078±0.740 a | 0.690 | 2.123±0.221 a | 0.502 | 1.546±0.060 b |
| Phosphorylethanolamine | 0.612± 0.063 a | 0.826 | 0.506± 0.091 a | 0.709 | 0.434± 0.100 a | 0.163±0.017 a | 1.548 | 0.252±0.055 a | 0.991 | 0.161±0.016 a |
| 9H-Purin-6-ol | 0.072± 0.015 b | 0.672 | 0.048± 0.004 b | 2.159 | 0.155± 0.028 a | 0.029±0.006 a | 0.730 | 0.021±0.004 b | 1.427 | 0.041±0.002 a |
| Dopamine | 0.614± 0.088 a | 0.964 | 0.592± 0.121 a | 0.517 | 0.318± 0.034 b | 0.092±0.015 c | 4.127 | 0.381±0.040 b | 7.474 | 0.691±0.085 a |
| N-Acetyl-D-glucosamine | 0.299± 0.072 a | 1.028 | 0.307± 0.047 a | 1.470 | 0.439± 0.029 a | 0.058±0.006 b | 3.008 | 0.173±0.020 a | 5.149 | 0.297± 0.068 a |
| Uric acid | ND | ND | ND | ND | ND | 0.288±0.022 b | 1.869 | 0.538±0.112 a | 0.391 | 0.113±0.014 b |
| Uridine | 2.168± 0.131 a | 0.718 | 1.556± 0.296 a | 0.787 | 1.707±0.103 a | 0.365±0.017 b | 2.607 | 0.950±0.153 a | 1.535 | 0.559±0.080 b |
| 2'-Deoxyinosine | 0.268± 0.049 b | 0.383 | 0.103± 0.018 b | 4.400 | 1.180± 0.103 a | 0.054±0.003 a | 1.072 | 0.057±0.007 a | 1.223 | 0.066±0.005 a |
| Inosine | 2.436± 0.140 a | 1.115 | 2.715± 0.090 a | 1.237 | 3.014± 0.453 a | 1.471±0.087 a | 0.943 | 1.387±0.106 a | 0.520 | 0.764±0.015 b |
| Adenosine | 2.299± 0.413 a | 1.434 | 3.296± 0.364 a | 0.673 | 1.546± 0.155 b | 0.602±0.032 c | 3.726 | 2.243±0.131 a | 2.196 | 1.321± 0.089 b |
| Cytidine | 0.463± 0.036 a | 0.307 | 0.142±0.070 b | 0.587 | 0.272± 0.038 b | 0.017±0.004 b | 9.791 | 0.164±0.006 a | 8.841 | 0.148±0.029 a |
| Guanosine | 1.857± 0.433 a | 1.178 | 2.188± 0.279 a | 1.162 | 2.158± 0.128 a | 0.304±0.018 b | 1.981 | 0.602±0.185 a | 2.344 | 0.712±0.097 a |
| **Lipidis and sterols** |  |  |  |  |  |  |  |  |  |  |
| Palmitelaidic acid | 0.107±0.004 b | 1.558 | 0.166± 0.016 b | 8.450 | 0.901± 0.129 a | 0.126±0.014 b | 1.541 | 0.195±0.013 a | 1.972 | 0.249±0.039 a |
| Palmitic Acid | 0.689±0.067 b | 1.356 | 0.935± 0.146 b | 3.880 | 2.675± 0.123 a | 0.378±0.026 b | 1.651 | 0.624±0.101 b | 3.339 | 1.261±0.191 a |
| Oleic Acid, (Z) | 0.263±0.029 b | 1.282 | 0.338± 0.047 b | 2.732 | 0.720± 0.092 a | 0.284±0.029 b | 1.162 | 0.330±0.061 a | 1.798 | 0.511±0.076 a |
| 11-Octadecenoic acid, (E) | 0.111±0.014 b | 1.463 | 0.163± 0.036 b | 6.790 | 0.756± 0.072 a | 0.049±0.008 b | 0.823 | 0.040±0.003 b | 1.792 | 0.088±0.016 a |
| Oleamide | 1.003±0.092 a | 0.775 | 0.777±0.103 b | 1.169 | 1.172± 0.046 a | 0.553±0.072 b | 1.363 | 0.754±0.057 a | 0.403 | 0.223± 0.014 c |
| 1-Monopalmitin | 0.138±0.022 b | 1.471 | 0.203±0.021 a | 1.518 | 0.209± 0.018 a | 0.070±0.003 b | 1.880 | 0.131±0.017 a | 1.749 | 0.122±0.020 a |
| 2-linoleoylglycerol | 0.311±0.046 c | 1.793 | 0.557± 0.078 b | 3.225 | 1.001± 0.067 a | 0.069±0.008 b | 9.024 | 0.619±0.045 a | 7.145 | 0.490±0.062 a |
| 1-Monolinolein | 0.037±0.006 b | 6.997 | 0.257± 0.030 a | 9.851 | 0.361±0.055 a | 0.008±0.001 b | 5.113 | 0.043±0.003 a | 4.580 | 0.038±0.009 a |
| 1-Monooleoylglycerol | 0.007±0.001 c | 2.225 | 0.016±0.001 b | 2.904 | 0.021±0.001 a | 0.014±0.002 b | 0.461 | 0.007±0.002 b | 1.776 | 0.025± 0.004 a |
| Glycerol monostearate | 0.091±0.009 c | 1.340 | 0.122± 0.005 b | 1.763 | 0.161±0.007 a | 0.051±0.008 b | 3.070 | 0.156±0.024 a | 1.484 | 0.076±0.010 b |
| Campesterol | 0.072±0.004 b | 1.286 | 0.092± 0.005 b | 1.889 | 0.135± 0.010 a | 0.009±0.001 a | 0.878 | 0.008±0.001 a | 0.896 | 0.008±0.002 a |
| Stigmasterol | 0.122±0.004 b | 2.320 | 0.282± 0.022 a | 2.427 | 0.295± 0.030 a | 0.182±0.017 a | 0.750 | 0.136±0.009 a | 0.941 | 0.171±0.028 a |
| Stigmast-5-en-3β-ol, (24S)- | 0.120±0.010 b | 1.281 | 0.153± 0.015 b | 2.329 | 0.278± 0.038 a | 0.173±0.020 a | 0.712 | 0.123±0.006 a | 0.883 | 0.153±0.027 a |
| **Others** |  |  |  |  |  |  |  |  |  |  |
| Boric acid | 2.033±0.210 a | 0.906 | 1.842± 0.229 a | 1.073 | 2.183±0.583 a | 0.517±0.013 b | 2.315 | 1.197±0.102 a | 1.342 | 0.694±0.079 b |
| Phosphoric acid | 8.975±1.178 a | 0.752 | 6.747± 0.389 a | 1.207 | 10.832± 2.091 a | 1.679±0.279 b | 2.864 | 4.809±0.736 a | 4.181 | 7.020±1.203 a |
| Urea | 4.864±1.023 a | 1.004 | 4.885±0.336 a | 1.068 | 5.196± 0.920 a | 0.788±0.139 b | 3.859 | 3.042±0.325 a | 3.795 | 2.991±0.588 a |
| Phosphoric acid | 18.390± 2.118 a | 0.918 | 16.888± 1.622 a | 0.869 | 15.975± 2.193 a | 3.115±0.314 c | 2.246 | 6.999±1.396 b | 3.413 | 10.632± 0.726 a |

Table showed GC-MS analysis of different metabolites from ‘TG’ and ‘SD’ roots of purslane cultivars at o, 100 mM and 200 mM stress, Metabolites with a twofold or more than two folds highlighted with red	square box AV and SE with ± and ND not detected different letters illustrated significance differences between treatments of detected metabolites, All values are ≤ 0.005.
